# Comparison of pneumonia incidence between long-acting muscarinic antagonist and inhaled corticosteroid plus long-acting beta agonist in patients with COPD

**DOI:** 10.1038/s41598-023-35223-3

**Published:** 2023-05-20

**Authors:** Eung Gu Lee, Youlim Kim, Yong Il Hwang, Kwang Ha Yoo, So Eun Lee, Kyung Yoon Jung, Doik Lee, Yong Bum Park, Chin Kook Rhee

**Affiliations:** 1 Division of Pulmonary and Critical Care Medicine, Department of Internal Medicine, Bucheon St. Mary’s Hospital, College of Medicine, The Catholic University of Korea, Seoul, South Korea; 2 Division of Pulmonary, Allergy and Critical Care Medicine, Department of Internal Medicine, Konkuk University Hospital, School of Medicine, Konkuk University, Seoul, South Korea; 3 Division of Pulmonary, Allergy and Critical Care Medicine, Department of Internal Medicine, Hallym University Sacred Heart Hospital, Anyang-Si, Gyeonggi-Do South Korea; 4 Medical Affairs, Boehringer-Ingelheim, Seoul, South Korea; 5 Real-World Solutions, IQVIA, Seoul, South Korea; 6 Division of Pulmonary, Allergy and Critical Care Medicine, Department of Internal Medicine, Hallym University Kangdong Sacred Heart Hospital, Seoul, South Korea; 7 Division of Pulmonary and Critical Care Medicine, Department of Internal Medicine, Seoul St. Mary’s Hospital, College of Medicine, The Catholic University of Korea, 222 Banpo-daero, Seocho-Gu, Seoul, 06591 South Korea

**Keywords:** Diseases, Medical research

## Abstract

Few studies have directly compared the incidence of pneumonia in patients on common chronic obstructive pulmonary disease (COPD) treatments such as long-acting muscarinic antagonists (LAMA) with those on inhaled corticosteroids and long-acting β_2_-agonist (ICS/LABA). Moreover, risk factors for pneumonia in COPD are still unclear. We aimed to compare the incidence of pneumonia in COPD patients on LAMA and those on ICS/LABA and explored the risk factors associated with pneumonia. This nationwide cohort study used Korean National Health Insurance claim data from January 2002 to April 2016. Patients who received COPD medication, either LAMA or ICS/LABA, with the COPD diagnostic code, were selected. We enrolled patients with good compliance (medication possession ratio ≥ 80%). The primary outcome was pneumonia in COPD patients initiating LAMA or ICS/LABA. We investigated the risk factors associated with pneumonia, including the sub-types of ICS treatments. After propensity score matching, the incidence rate per 1000 person-years of pneumonia was 93.96 for LAMA (n = 1003) and 136.42 for ICS/LABA (n = 1003) patients (p < 0.001). The adjusted hazard ratio (HR) for pneumonia in patients on fluticasone/LABA was 1.496 (95% confidence interval [CI] 1.204–1.859) compared with LAMA (p < 0.001). In multivariable analysis, a history of pneumonia was a risk factor associated with pneumonia (HR 2.123; 95% CI 1.580–2.852; p < 0.001). The incidence of pneumonia was higher in COPD patients on ICS/LABA compared with those on LAMA. It is recommended that ICS use be avoided in COPD patients with high pneumonia risk.

## Introduction

Chronic obstructive pulmonary disease (COPD) is a progressive chronic disease characterized by persistent respiratory symptoms and airflow limitation^1^. According to the Global Burden of Disease, COPD was the third leading cause of death worldwide in 2010^2^. Pharmacotherapy and hospitalization due to severe disease, exacerbations, and comorbidities contribute to the economic cost of COPD^3^. Therefore, appropriate medication selection can help control these factors and reduce both individual and societal costs.

Inhaled bronchodilators, such as long-acting muscarinic antagonists (LAMA) and long-acting β_2_-agonists (LABA) are regularly administered to reduce COPD symptoms while preventing progression and exacerbation^1^. Compared to monotherapy, fixed-dose combinations of LAMA and LABA within a single inhaler show improved lung function, symptom reduction, and overall improvement in quality of life^4^. The combination of LAMA/LABA with inhaled corticosteroids (ICS) is recommended for patients with severe COPD and frequent exacerbations^1^. An increased blood eosinophil count in COPD is associated with a higher exacerbation rate and favorable response to ICS^5,6^. The Global Initiative for Chronic Obstructive Lung Disease (GOLD) 2019 initially recommended that, for patients in GOLD group D, an eosinophil count of ≥ 300 cells/μL was an indicator for ICS/LABA treatment, with this threshold identifying patients more likely to benefit from ICS treatment^7^.

ICS increase the risk of side effects such as oropharyngeal candidiasis, hoarse voice, skin bruising, tuberculosis, and pneumonia^8,9^; also, physicians should remain vigilant for pneumonia development in COPD patients on ICS-containing regimens^10,11^. However, in clinical practice, ICS is overused without due consideration of the risks and benefits^12^.

Randomized controlled trials do not always reflect the entire population affected by a disease. Compared to the general COPD population, COPD trial participants often have fewer comorbidities, lower age, and milder disease. There is already another study comparing the risk of pneumonia requiring hospitalization when patients with chronic airway disease used ICS/LABA in a real-world setting, using claims data from the National Health Insurance Service (NHIS) in South Korea^13^. However, there is a limitation in those patients with all chronic airway diseases such as COPD, asthma, bronchiectasis, and tuberculosis-destroyed lung were targeted, and the pneumonia risk was analyzed by dividing ICS/LABA by device—pressurized metered-dose inhalers (pMDIs) and dry powder inhalers (DPIs)—rather than a component. The objectives of our study were to compare the incidence and the risk of pneumonia between ICS/LABA therapy and LAMA monotherapy in COPD patients in the real-world.

## Methods

### Study design

This was a retrospective, observational, cohort study using claims data from the National Health Insurance Service (NHIS) to compare effectiveness and safety outcomes in COPD patients commencing LAMA or ICS/LABA treatment in South Korea. The study protocol followed the principles of the Declaration of Helsinki and was approved by the Institutional Review Board (IRB) of Konkuk University Medical Center (IRB No.: KUMC2020-06-013).

**Figure 1:**
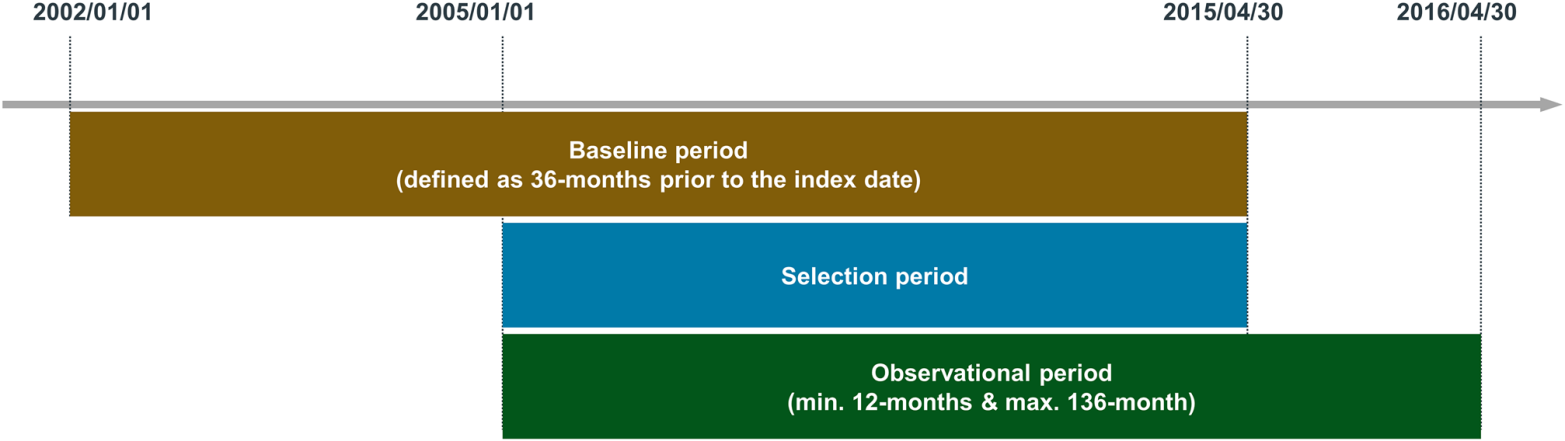
Overview of the study design.

Patients receiving LAMA or ICS/LABA, with the COPD diagnostic code, between 1 January 2005 and 30 April 2015, were selected as the study group. The index date was the first prescription date of LAMA or ICS/LABA with the COPD diagnosis on record. A schematic diagram of the study design is shown in Fig. 1.

### Inclusion criteria

**Figure 2:**
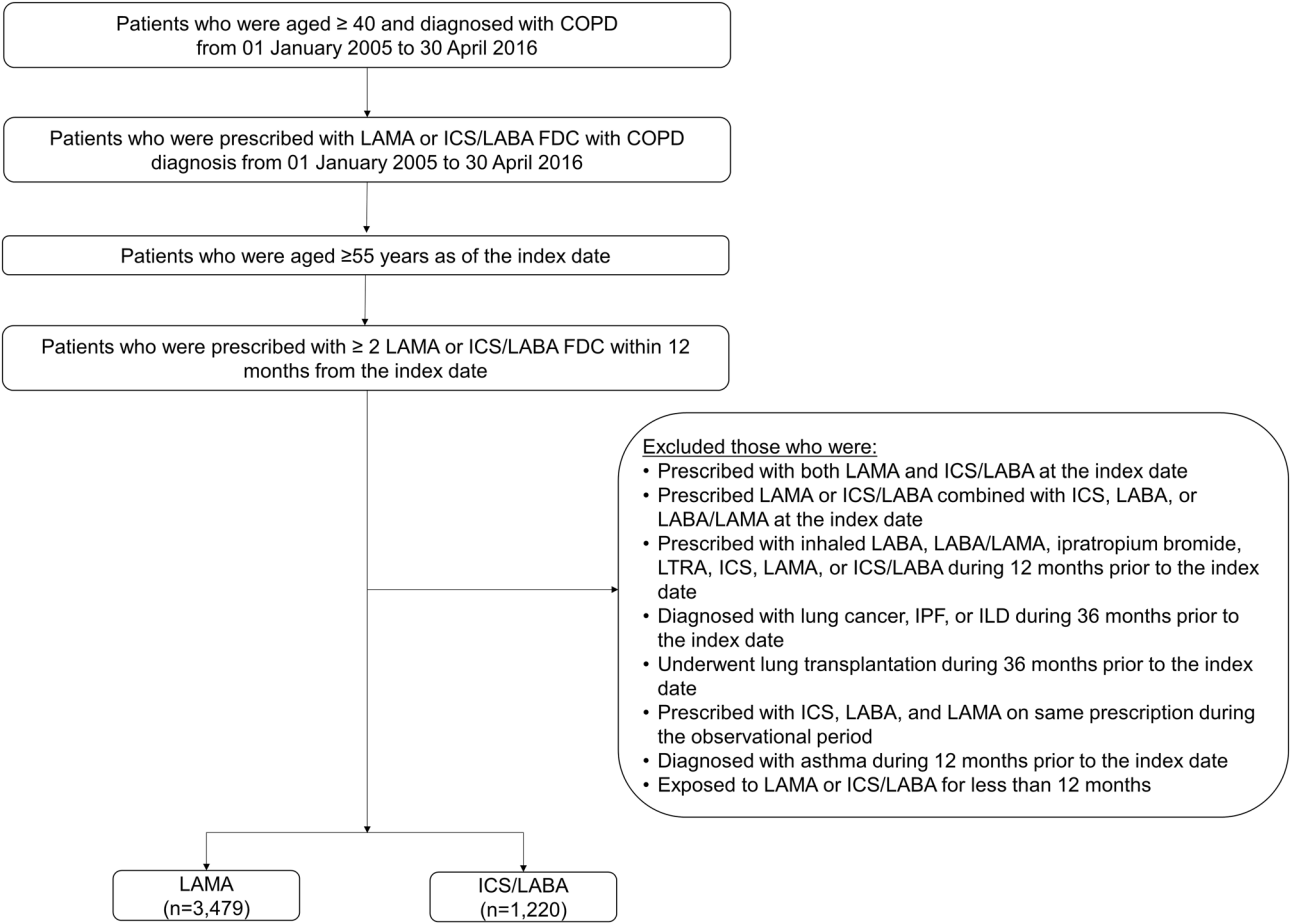
Flow chart of patient selection.

To increase the likelihood of a COPD diagnosis, we only included patients aged ≥ 55 years on the index date; other inclusion criteria were two or more inpatient or outpatient claims for LAMA monotherapy or ICS/LABA fixed dose combination (FDC) with the International Classification of Disease-10 (ICD-10) code for COPD (J43.x–44.x [except J43.0]) recorded as any diagnosis in inpatient claims or primary to 4th secondary diagnosis in outpatient claims, and prescription of LAMA or ICS/LABA more than twice within 12 months of the index date (Fig. 2).

### Exclusion criteria

Patients were excluded if prescribed LAMA and ICS/LABA; LABA or LAMA/LABA at the index date; ipratropium; leukotriene receptor antagonist; or ICS. Also excluded were patients diagnosed with lung cancer, interstitial lung disease, or lung transplantation during the baseline period prior to the index date (Fig. 2).

### Outcomes

The primary outcome was pneumonia in COPD patients commencing LAMA or ICS/LABA. Pneumonia was defined as one or more inpatient or outpatient claims with (1) ICD-10 codes for pneumonia recorded as any diagnosis in inpatient claims or primary to 4th secondary diagnosis in outpatient claims; (2) diagnostic test codes for chest X-ray or computed tomography; and (3) antibiotic prescription after the index date.

### Statistical analysis

The Chi-squared test was used for categorical variables and the *t*-test or Wilcoxon rank-sum test for continuous variables, to compare baseline characteristics between treatment groups. Hazard ratios (HRs) with 95% confidence intervals (CIs) were estimated using Cox-proportional hazards regression to compare the risk of primary outcomes between the study groups. All HRs were estimated using the LAMA group as a reference.

Propensity score (PS) matching was used to reduce confounding factors and to balance comparability between the study groups since there was a possibility that initial treatment could be based on patient demographics and baseline characteristics. 1:1 PS matching used age, sex, economic status, exacerbation history, pneumonia, comorbidities, and index year as co-variables. Significance was set at 0.05, two tailed. Software used for statistical analysis was SAS^®^ 9.4 (SAS Institute, Cary, North Carolina, USA).

### Ethics approval and consent to participate

The study protocol followed the principles of the Declaration of Helsinki and was approved by the Institutional Review Board (IRB) of Konkuk University Medical Center (IRB No.: KUMC2020-06-013). The requirement for informed consent from the participants was waived by the IRB of Konkuk University Medical Center (IRB No.: KUMC2020-06-013) due to the retrospective nature of this study.

## Results

### Baseline characteristics

The cohort included 4699 patients, 3479 and 1220 of whom received LAMA and ICS/LABA, respectively. After PS matching, the cohort included 2006 patients, with 1003 patients in each group. The LAMA group received either tiotropium, aclidinium, or glycopyrronium bromide. The ICS/LABA group received either fluticasone propionate/salmeterol, fluticasone propionate/formoterol, fluticasone furoate/vilanterol, beclometasone/formoterol or budesonide/formoterol.

After PS matching, the characteristics of the two groups (Table 1) showed good overall balance as each absolute standardized difference (ASD) was < 0.1. The observation period was significantly longer in the LAMA group (750.23 ± 670.21 days vs. 604.27 ± 476.13 days, p < 0.001).

**Table 1:** Baseline characteristics of enrolled patients.

Characteristics	Unmatched population								Propensity score-matched population							
	All (n = 4699)		LAMA (n = 3479)		ICS/LABA (n = 1220)		p-value	ASD	All (n = 2006)		LAMA (n = 1003)		ICS/LABA (n = 1003)		p-value	ASD
	n	%	n	%	n	%			n	%	n	%	n	%		
Observation period (days)																
Mean ± SD	700.04	574.80	725.4	590.93	627.72	519.56	< 0.001		677.25	585.75	750.23	670.21	604.27	476.13	< 0.001	
Age (years)																
Mean ± SD	69.63	7.91	69.62	7.85	69.67	8.08	0.87	0.01	69.57	7.97	69.73	7.77	69.4	8.17	0.29	0.04
Median	69		69		69				69		70		69			
Min	55		55		55				55		55		55			
Max	102		102		94				98		98		94			
P25	64		64		64				64		64		63			
P75	75		75		76				75		75		75			
55 to < 75	3375	0.72	2513	0.72	862	0.71	0.29	0.05	1424	0.71	711	0.71	713	0.71	0.92	0.08
75+	1324	0.28	966	0.28	358	0.29			582	0.29	292	0.29	290	0.29		
Sex																
Male	3889	0.83	2991	0.86	898	0.74	< 0.001	0.31	1531	0.76	771	0.77	760	0.76	0.56	0.03
Female	810	0.17	488	0.14	322	0.26			475	0.24	232	0.23	243	0.24		
Income level																
1st quartile	686	0.15	513	0.15	173	0.14	< 0.001	0.41	326	0.16	167	0.17	159	0.16	0.97	0.03
2nd quartile	651	0.14	509	0.15	142	0.12			254	0.13	125	0.12	129	0.13		
3rd quartile	928	0.20	733	0.21	195	0.16			347	0.17	176	0.18	171	0.17		
4th quartile	1653	0.35	1290	0.37	363	0.30			677	0.34	338	0.34	339	0.34		
Medical aid	781	0.17	434	0.12	347	0.28			402	0.20	197	0.20	205	0.20		
Hospital type																
General hospital	4036	0.86	3024	0.87	1012	0.83	< 0.001	0.15	1707	0.85	860	0.86	847	0.84	0.78	0.05
Hospital	251	0.05	174	0.05	77	0.06			134	0.07	62	0.06	72	0.07		
Clinic	394	0.08	276	0.08	118	0.10			158	0.08	77	0.08	81	0.08		
Others	18	0.00	5	0.00	13	0.01			7	0.00	4	0.00	3	0.00		
History of COPD exacerbation																
None	3956	0.84	2930	0.84	1026	0.84	0.91	0.01	1651	0.82	824	0.82	827	0.82	0.66	0.04
1 moderate	320	0.07	239	0.07	81	0.07			145	0.07	69	0.07	76	0.08		
≥ 2 moderate OR ≥ 1 severe	423	0.09	310	0.09	113	0.09			210	0.10	110	0.11	100	0.10		
History of pneumonia																
No	4238	0.90	3113	0.89	1125	0.92	< 0.001	0.09	1831	0.91	917	0.91	914	0.91	0.81	0.01
Yes	461	0.10	366	0.11	95	0.08			175	0.09	86	0.09	89	0.09		
mCCI																
Mean ± SD	1.81	1.94	1.85	1.93	1.67	1.96	< 0.001	0.09	1.87	2.05	1.87	2.12	1.87	1.99	0.61	0.00
Median	1								1		1		1			
Min	0								0		0		0			
Max	15								15		15		13			
P25	0								0		0		0			
P75	3								3		3		3			
0.1	2694	0.57	1946	0.56	748	0.61	< 0.001	0.12	1150	0.57	582	0.58	568	0.57	0.79	0.04
2	567	0.12	436	0.13	131	0.11			236	0.12	113	0.11	123	0.12		
3	780	0.17	608	0.17	172	0.14			312	0.16	159	0.16	153	0.15		
4+	658	0.14	489	0.14	169	0.14			308	0.15	149	0.15	159	0.16		
mCCI category																
Congestive heart failure	459	0.10	345	0.10	114	0.09	0.56	0.02	207	0.10	99	0.10	108	0.11	0.51	0.03
Dementia	165	0.04	128	0.04	37	0.03	0.29	0.04	70	0.03	33	0.03	37	0.04	0.63	0.02
Chronic pulmonary disease	2613	0.56	2003	0.58	610	0.50	< 0.001	0.15	1117	0.56	563	0.56	554	0.55	0.69	0.02
Rheumatologic disease	171	0.04	115	0.03	56	0.05	0.04	0.07	103	0.05	50	0.05	53	0.05	0.76	0.02
Mild liver disease	979	0.21	743	0.21	236	0.19	0.14	0.05	422	0.21	205	0.20	217	0.22	0.51	0.03
Diabetes with chronic complications	471	0.10	354	0.10	117	0.10	0.56	0.02	203	0.10	93	0.09	110	0.11	0.21	0.06
Hemiplegia or paraplegia	52	0.01	41	0.01	11	0.01	0.43	0.03	20	0.01	9	0.01	11	0.01	0.65	0.02
Renal disease	130	0.03	91	0.03	39	0.03	0.29	0.03	76	0.04	39	0.04	37	0.04	0.82	0.01
Any malignancy, including lymphoma and leukemia	630	0.13	484	0.14	146	0.12	0.09	0.06	267	0.13	136	0.14	131	0.13	0.74	0.01
Moderate or severe liver disease	41	0.01	30	0.01	11	0.01	0.90	0.00	23	0.01	12	0.01	11	0.01	0.83	0.01
Metastatic solid tumor	60	0.01	46	0.01	14	0.01	0.64	0.02	32	0.02	20	0.02	12	0.01	0.15	0.07
HIV	1	0.00	0	0.00	1	0.00	0.09	NA	0	0.00	0	0.00	0	0.00	NA	NA
Index year																
2005	361	0.08	77	0.02	285	0.23	< 0.001	0.75	161	0.08	77	0.08	84	0.08	0.99	0.07
2006	306	0.07	185	0.05	121	0.10			230	0.11	123	0.12	107	0.11		
2007	363	0.08	258	0.07	105	0.09			212	0.11	107	0.11	105	0.10		
2008	453	0.10	374	0.11	79	0.06			158	0.08	79	0.08	79	0.08		
2009	490	0.10	379	0.11	111	0.09			220	0.11	110	0.11	110	0.11		
2010	585	0.12	481	0.14	104	0.09			202	0.10	98	0.10	104	0.10		
2011	566	0.12	461	0.13	105	0.09			214	0.11	110	0.11	104	0.10		
2012	545	0.12	441	0.13	104	0.09			208	0.10	104	0.10	104	0.10		
2013	447	0.10	363	0.10	84	0.07			159	0.08	75	0.07	84	0.08		
2014	465	0.10	369	0.11	96	0.08			191	0.10	95	0.09	96	0.10		
2015	117	0.02	91	0.03	26	0.02			51	0.03	25	0.02	26	0.03		

*LAMA* Long-acting muscarinic antagonists, *ICS* Inhaled corticosteroid, *LABA* Long acting β2-agonists, *ASD* Absolute standardized difference, *SD* Standard deviation, *COPD* Chronic obstructive pulmonary disease, *mCCI* Modified Charlson comorbidity index, *HIV* Human immunodeficiency virus.

### Pneumonia incidence rate

Patients who received ICS/LABA had a higher pneumonia incidence rate than those who received LAMA (93.96/1000 PYs vs. 136.42/1000 PYs, p = 0.0004).

The pneumonia incidence rate was higher in ICS/LABA compared to LAMA in the 55–74 years age group (p = 0.0013) and in the group with no history of exacerbation (p = 0.0029) or one moderate exacerbation (p = 0.0116) (Table 2). The incidence rate of pneumonia-related hospitalization was also significantly higher with ICS/LABA compared with LAMA (p = 0.0001) (Additional file 1; Supplementary Table S1).

**Table 2:** Incidence rate of pneumonia.

Pneumonia	Unmatched population				Propensity score-matched population			
	All	LAMA	ICS/LABA	p-value	All	LAMA	ICS/LABA	p-value
Incidence rate per 1000 PYs	102.78	90.20	147.09	< 0.001	112.41	93.96	136.42	< 0.001
Patients with event	812	555	257		366	173	193	
Sum of PYs	7900.29	6153.06	1747.23		3256.03	1841.3	1414.73	
Time to event								
Mean	432.61	446.42	402.79		441.61	523.64	368.08	
SD	548.35	563.61	513.63		568.5	675.18	441.04	
Median	257.5	256	265		265.5	280	259	
Min	2	2	2		2	2	2	
Max	3481	3481	2995		3481	3481	2791	
P25	96	96	93		103	112	98	
P75	495	525	471		514	604	437	
Age (years)								
55 to < 75	90.35	77.90	136.62	< 0.001	100.45	82.37	125.05	0.002
75+	137.84	126.49	172.47	0.018	143.86	126.66	163.82	0.144
Sex								
Male	100.99	89.96	146.01	< 0.001	114.73	97.85	136.73	0.005
Female	111.66	91.67	150.17	0.003	104.59	80.80	135.40	0.023
History of COPD exacerbation								
None	95.27	82.78	139.34	< 0.001	105.25	88.78	126.5	0.003
1 moderate	140.57	122.65	214.09	0.045	142.28	84.91	220.8	0.012
≥ 2 moderate OR ≥ 1 severe	149.54	140.35	178.36	0.277	149.82	137.98	165.74	0.506

*LAMA* long-acting muscarinic antagonists, *ICS* inhaled corticosteroid, *LABA* long-acting β2-agonists, *PYs* Person years, *SD* standard deviation, *COPD* Chronic obstructive pulmonary disease.

There were no significant differences in the incidence rate of outpatient pneumonia and pneumonia-related death between the patients on LAMA and ICS/LABA according to age groups, sex, and higher exacerbation history (Additional file 1; Supplementary Tables S2 and S3).

### Frequency of pneumonia

After PS matching, the frequency of pneumonia events showed no significant difference between LAMA and ICS/LABA (p = 0.172) (Additional file 1; Supplementary Table S4). The frequency of pneumonia-related hospitalization events was higher in the ICS/LABA group than in the LAMA group (Additional file 1; Supplementary Table S5). The frequency of outpatient pneumonia events was not significantly different between LAMA and ICS/LABA (Additional file 1; Supplementary Table S6).

### Risk of pneumonia

Treatment with ICS/LABA compared with LAMA was associated with a higher risk of pneumonia (HR 1.374; 95% CI 1.116–1.692; p = 0.0028; Additional file 1, Supplementary Table S7). This also applied in the 55 to < 75 years age group (p = 0.0126), males (p = 0.0187), patients treated in general hospitals (hospitals with > 100 beds with internal medicine specialists, p = 0.0122), patients with no history of COPD exacerbation (p = 0.0150) or a history of one moderate exacerbation (p = 0.0050), and patients with no prior history of pneumonia (p = 0.0010).

A higher proportion of patients on ICS/LABA were hospitalized with pneumonia events compared with LAMA (HR 1.610; 95% CI 1.234–2.101; p = 0.0005; Additional file 1, Supplementary Table S8), and this applied irrespective of age group. The risk was significantly higher in ICS/LABA patients in the lower quartile of income (p = 0.0081), and those who had never experienced COPD exacerbation (p = 0.0021) or had experienced one moderate exacerbation (p = 0.0168).

There were no significant differences in outpatient pneumonia events and pneumonia-related deaths between ICS/LABA and LAMA (Additional file 1; Supplementary Tables S9 and S10).

### Pneumonia probability

**Figure 3:**
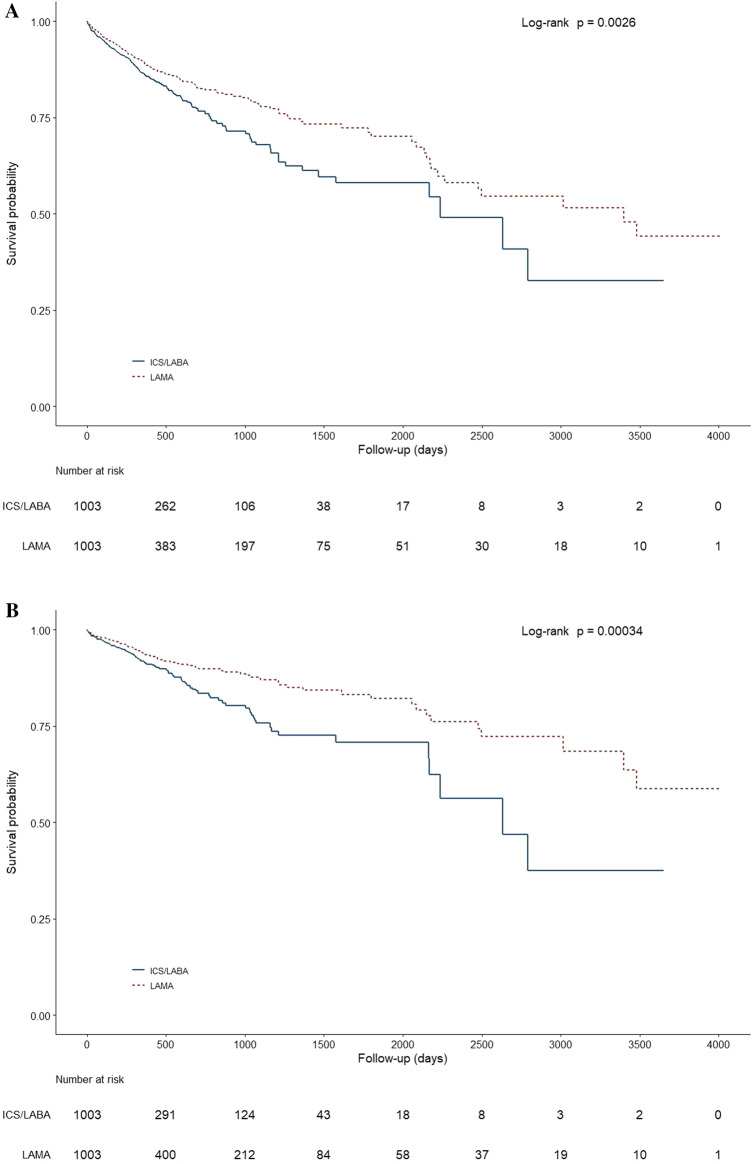

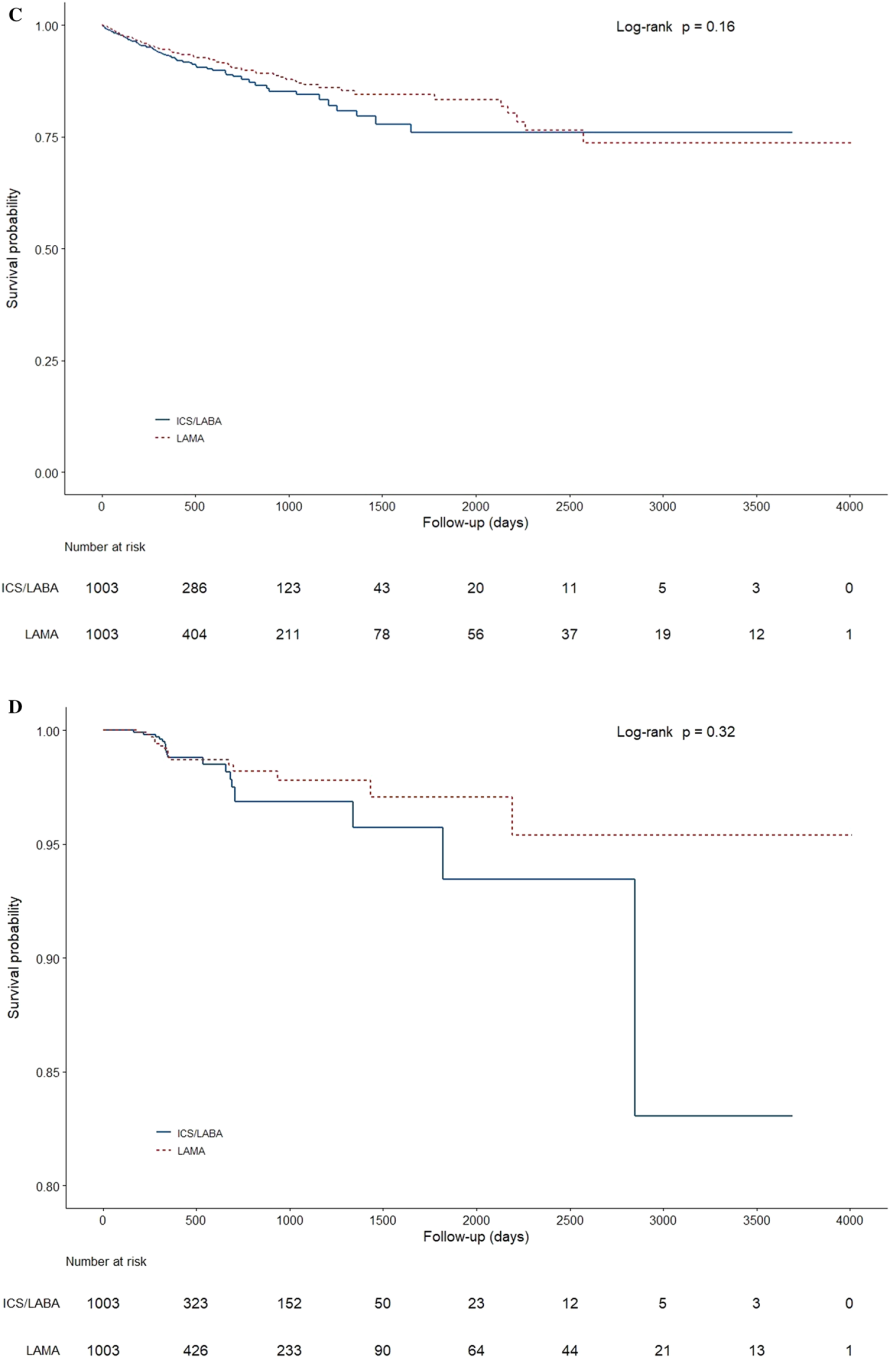
(**A**) Time to first pneumonia event in patients on ICS/LABA vs. LAMA. (**B**) Time to first pneumonia-related hospitalization event in patients on ICS/LABA vs. LAMA. (**C**) Time to first outpatient pneumonia event in patients on ICS/LABA vs. LAMA. (**D**) Time to pneumonia-related death in patients on ICS/LABA vs. LAMA. *ICS* inhaled corticosteroids, *LABA* long acting β_2_-agonists, *LAMA* long-acting muscarinic antagonists.

Kaplan–Meier probability estimates revealed that the risk of pneumonia, based on the time to first pneumonia events, was significantly higher in ICS/LABA (p = 0.0026; Fig. 3A). The risk of pneumonia-related hospitalization was also significantly higher in ICS/LABA (p = 0.00034; Fig. 3B). However, Kaplan–Meier probability estimates showed no significant differences in the risks of outpatient pneumonia events or pneumonia-related deaths between the two groups (Fig. 3C,D).

### Risk factors associated with pneumonia

In the PS-matched population, ICS/LABA treatment was a risk factor associated with pneumonia (HR 1.389; 95% CI 1.127–1.713; p = 0.0021; Table 3). A secondary analysis was performed on patients on ICS/LABA per ICS treatment. In patients using fluticasone, ICS/LABA was a risk factor associated with pneumonia (HR 1.496; 95% CI 1.204–1.859; p = 0.0003). However, in patients on non-fluticasone ICS/LABA, ICS/LABA was not a risk factor associated with pneumonia (HR 1.002; 95% CI 0.682–1.473; p = 0.9914). In COPD patients, another significant pneumonia-associated risk factor was a history of pneumonia (HR 2.123; 95% CI 1.580–2.852; p < 0.0001).

**Table 3:** Pneumonia-associated risk factors.

	Unmatched population						Propensity score-matched population					
	Crude HR			Adjusted HR			Crude HR			Adjusted HR		
	HR	95% CI	p-value	HR	95% CI	p-value	HR	95% CI	p-value	HR	95% CI	p-value
Index med												
LAMA	Ref	–	–	Ref	–	–	Ref	–	–	Ref	–	–
ICS/LABA with fluticasone	1.622	1.384–1.901	< 0.001	1.635	1.388–1.926	< 0.001	1.318	0.999–1.739	0.051	1.496	1.204–1.859	< 0.001
ICS/LABA without fluticasone	1.277	0.942–1.732	0.115	1.232	0.906–1.675	0.184	1.120	0.653–1.921	0.680	1.002	0.682–1.473	0.991
ICS/LABA	1.550	1.336–1.798	< 0.001	1.551	1.331–1.809	< 0.001	1.374	1.116–1.692	0.003	1.389	1.127–1.713	0.002
Age (years)												
55 to < 75	Ref	–	–	Ref	–	–	Ref	–	–			
75+	1.495	1.294–1.727	< 0.001	1.431	1.235–1.657	< 0.001	0.914	0.630–1.326	0.635			
Sex												
Male	0.912	0.763–1.090	0.3095				0.727	0.430–1.230	0.2352			
Female	Ref	–	–				Ref	–	–			
Income level												
1st quartile	Ref	–	–	Ref	–	–	Ref	–	–			
2nd quartile	1.168	0.893–1.528	0.257	1.175	0.898–1.537	0.241	1.279	0.623–2.626	0.503			
3rd quartile	1.118	0.869–1.439	0.385	1.140	0.886–1.468	0.309	1.048	0.532–2.062	0.893			
4th quartile	1.185	0.944–1.488	0.144	1.150	0.914–1.446	0.234	0.950	0.508–1.776	0.873			
Medical aid	1.575	1.235–2.010	< 0.001	1.464	1.143–1.875	0.003	1.005	0.511–1.977	0.988			
Hospital type												
General hospital	1.400	1.073–1.828	0.013	1.337	1.022–1.751	0.034	1.844	0.902–3.767	0.093	1.331	0.885–2.003	0.170
Hospital	1.284	0.864–1.907	0.217	1.117	0.751–1.661	0.586	0.821	0.291–2.315	0.709	0.823	0.449–1.511	0.5305
Clinic	Ref	–	–	Ref	–	–	Ref	–	–	Ref	–	–
Others	0.908	0.222–3.713	0.8934	0.640	0.156–2.632	0.536	1.230	0.065–23.221	0.890	1.182	0.18–9.867	0.8695
History of pneumonia												
No	Ref	–	–	Ref	–	–	Ref	–	–	Ref	–	–
Yes	2.479	2.074–2.963	< 0.0001	2.400	1.978–2.911	< 0.001	2.333	1.274–4.272	0.006	2.123	1.580–2.852	< 0.0001
History of COPD exacerbation												
None	Ref	–	–	Ref	–	–	Ref	–	–			
1 moderate	1.429	1.111–1.839	0.006	1.240	0.950–1.619	0.114	1.302	0.888–1.909	0.177			
≥ 2 moderate OR ≥ 1 severe	1.574	1.279–1.936	< 0.0001	1.083	0.860–1.364	0.496	1.446	1.079–1.939	0.014			
mCCI												
0, 1	Ref	–	–	Ref	–	–	Ref	–	–			
2	0.793	0.623–1.009	0.060	0.815	0.639–1.040	0.010	1.051	0.578–1.913	0.870			
3	1.064	0.881–1.283	0.521	0.964	0.792–1.174	0.717	0.972	0.552–1.710	0.920			
4+	1.230	1.012–1.496	0.038	1.007	0.818–1.240	0.950	0.871	0.533–1.421	0.580			
mCCI category												
Congestive heart failure												
No	Ref	–	–				Ref	–	–			
Yes	1.053	0.839–1.322	0.655				0.885	0.505–1.551	0.669			
Dementia												
No	Ref	–	–				Ref	–	–			
Yes	1.160	0.805–1.671	0.425				1.000	0.375–2.664	1.000			
Chronic pulmonary disease												
No	Ref	–	–	Ref	–	–	Ref	–	–			
Yes	1.223	1.063–1.407	0.005	1.106	0.939–1.302	0.2273	1.160	0.795–1.693	0.442			
Rheumatologic disease												
No	Ref	–	–				Ref	–	–			
Yes	1.232	0.878–1.730	0.229				0.917	0.405–2.078	0.835			
Mild liver disease												
No	Ref	–	–				Ref	–	–			
Yes	1.058	0.895–1.250	0.510				0.814	0.521–1.272	0.366			
Diabetes with chronic complications												
No	Ref	–	–				Ref	–	–			
Yes	1.037	0.825–1.303	0.510				0.724	0.413–1.270	0.260			
Hemiplegia or paraplegia												
No	Ref	–	–				Ref	–	–			
Yes	1.112	0.576–2.145	0.752				1.500	0.251–8.977	0.657			
Renal disease												
No	Ref	–	–				Ref	–	–			
Yes	1.021	0.655–1.592	0.9257				1.125	0.434–2.915	0.809			
Any malignancy, including lymphoma and leukemia												
No	Ref	–	–				Ref	–	–			
Yes	1.032	0.843–1.264	0.759				1.226	0.763–1.970	0.400			
Moderate or severe liver disease												
No	Ref	–	–	Ref	–	–	Ref	–	–			
Yes	1.985	1.095–3.601	0.024	1.916	1.030–3.567	0.0401	1.667	0.398–6.974	0.484			
Metastatic solid tumor												
No	Ref	–	–				Ref	–	–			
Yes	1.055	0.582–1.912	0.860				1.834	0.254–2.732	0.764			
HIV												
No	Ref	–	–				NA	NA	NA			
Yes	NA	NA	NA				NA	NA	NA			

*HR* hazard ratio, *CI* Confidence interval, *COPD* chronic obstructive pulmonary disease, *mCCI* modified Charlson comorbidity index, *HIV* human immunodeficiency virus, *NA* not applicable.

## Discussion

In this study, we compared the risk of pneumonia associated with long-term ICS/LABA or LAMA treatment for COPD and found that the overall risk of pneumonia was significantly higher in ICS/LABA treatment. The incidence rates of pneumonia and pneumonia-related hospitalization were higher in patients on ICS/LABA, especially in the youngest included age group (55 to < 75 years). This trend was even observed in COPD patients with COPD exacerbation history (no or one moderate exacerbation).

Additionally, the subgroups with higher pneumonia risk on ICS/LABA—compared to LAMA—were those with no history of pneumonia; treatment at a hospital-level medical institution with inpatient beds rather than a primary medical institution such as primary care, or a lower income class in the 4th quartile. Regarding comorbidities, pneumonia risk was higher when ICS/LABA was used in patients with chronic pulmonary diseases such as bronchiectasis and TB-destroyed lungs resulting in cough, sputum, and dyspnea.

In the TORCH study, fluticasone propionate with long-acting β_2_-agonists salmeterol vs. placebo showed an increased risk of pneumonia (HR 1.64; 95% CI 1.33–2.02; p < 0.001), a similar effect to fluticasone propionate alone (HR 1.53; 95% CI 1.24–1.89; p < 0.001), contrasting with salmeterol alone (HR 1.09; 95% CI 0.87–1.37, p = 0.465)^10^. More patients using an ICS-containing regimen had severe pneumonia compared to either salmeterol or placebo.

In the INSPIRE study, the patient group treated with fluticasone propionate/salmeterol showed a significantly higher risk of pneumonia than those treated with tiotropium (HR 1.94; 95% CI 1.19–3.17, p = 0.008)^14^. This is consistent with our results that ICS increases the risk of pneumonia compared to patients who used LAMA. However, the INSPIRE study is limited by the protocol’s unclear prior definition due to the lack of prediction of excessive occurrence of pneumonia during treatment.

In the post hoc analysis of the 4-year UPLIFT^®^ trial, COPD patients who used fluticasone propionate had a higher risk of pneumonia than patients who did not use ICS (HR 1.33; 95% CI 1.00–1.75; p = 0.046)^15^. The risk of pneumonia-related hospitalization was also greater in patients treated with fluticasone propionate compared with other or no ICS. However, there was no significant difference in pneumonia and pneumonia-related hospitalization risk between patients treated with ICS other than fluticasone and those without ICS^15^. This suggests that fluticasone is associated with pneumonia more than any other ICS.

In the PATHOS study, the rate of pneumonia and admission to hospital and mortality related to pneumonia were higher in patients treated with fluticasone/salmeterol compared with budesonide/formoterol^16^. Our analysis, based on real-world data, had results consistent with the above clinical trial. The use of ICS/LABA was also a risk factor associated with pneumonia. When ICS/LABA was divided into a group containing fluticasone and a group containing ICS other than fluticasone, only ICS/LABA with fluticasone was a risk factor for pneumonia.

Yang et al. performed a meta-analysis of 25 double-blind clinical trials (including 49,982 patients) using ICS as an intervention drug and non-ICS treatment as a control group^17^. ICS treatment was significantly associated with an increased risk of pneumonia in COPD patients. In a subgroup analysis based on ICS type, fluticasone increased the risk of pneumonia regardless of high-, medium-, or low-doses. Contrastingly, budesonide did not increase the risk of pneumonia, irrespective of dose^17^.

A study based on real-world clinical practice from the UK's Clinical Practice Research Datalink compared the efficacy and safety of treatment with ICS/LABA or LAMA in COPD patients aged > 55 years from 2002 to 2015^18^. ICS/LABA was associated with a higher risk of pneumonia than LAMA and showed an exceptionally higher risk when fluticasone was used as ICS. In a real-world, observational study comparing the efficacy and safety of ICS/LABA and a LABA/LAMA combination instead of LAMA monotherapy, the incidence of pneumonia was also high when ICS/LABA was used^19^. However, the study did not analyze the pneumonia risk according to the type of ICS.

Lee et al. also analyzed the relationship between ICS and pneumonia risk in patients with COPD using data from the Korean NHIS^20^. In this study, all patients were classified into ICS and non-ICS users based only on the inclusion of ICS, regardless of the ingredients of the inhaler. Pneumonia was significantly related to ICS use, and the pneumonia risk increased as the cumulative dose of ICS increased. However, the daily dose of ICS was not associated with pneumonia^20^. And, as in other studies, fluticasone propionate (HR 1.79; 95% CI 1.70–1.89; p < 0.0001) and fluticasone furoate (HR 1.80; 95% CI 1.61–2.01; p < 0.0001) showed a higher risk of pneumonia than other ICS such as budesonide (HR 1.44; 95% CI 1.35–1.54; p < 0.0001).

It is also true that ICS, particularly fluticasone, makes a significant difference in lowering acute exacerbations in COPD patients. In the IMPACT trial, once-daily single-inhaler triple therapy with fluticasone furoate/umeclidinium/vilanterol resulted in a significantly lower rate of moderate or severe COPD exacerbations and better lung function and health-related quality of life than dual therapy with fluticasone furoate/vilanterol or the dual bronchodilator umeclidinium/vilanterol among patients with symptomatic COPD and a history of exacerbations^21^. Although the incidence of AE COPD is almost ten times higher than that of pneumonia, the GOLD strategy currently discourages using ICS in patients with repeated pneumonia episodes or blood eosinophil counts less than 100 cells/μL or a history of mycobacterial infection. Therefore, the decision to use ICS in patients with COPD must always be made carefully, and caution is required during use.

Several studies, including ours, showed that fluticasone increased the risk of pneumonia even more so than other ICS^16,22^. This may be caused by fluticasone's lipid-solubility and slow dissolution properties, implying that it remains in the airway lining fluid longer compared to other ICS causing local immunosuppression^16,22^.

Our study had several strengths compared to other observational studies in a real-world setting. The analysis was performed using a nationwide population-based cohort, the NHID from the NHIS (including 98% of the population of South Korea) and had a long-term observational period. Pneumonia, pneumonia-related hospitalization, outpatient pneumonia, and pneumonia-related death were systematically analyzed according to risk by incidence rate, frequency, hazard ratio, and probability estimates based on time to the first event. Compared to LAMA, subgroups showing high pneumonia risk among patients using ICS/LABA were analyzed. These subgroup analyses included age; sex; income level; COPD risk group based on exacerbation history; history of pneumonia; and comorbidities. The analysis of these high-risk group, large sample size, and longer duration is novel and has not been seen in previous studies.

There were several limitations to our study. First, considering the inclusion and exclusion criteria, the sample does not represent the entire COPD population. Second, the data used are not very recent. Third, there is a lack of rationale regarding the criteria for prescribing ICS/LABA or LAMA. Fourth, because our study was based on NHIS data, pneumonia events were identified only by diagnostic codes, and radiologic findings or severity analyses were not performed. Fifth, the study could not include a control group of non-treatment subjects. Sixth, no analysis was performed on the probability of pneumonia by comparing ICS/LABA with and without fluticasone using the Kaplan–Meier curve, nor the frequency of use of each type of ICS or the pneumonia risk for each ICS type other than fluticasone. Finally, we did not analyze the pneumonia risk according to the dose of ICS.

## Conclusions

The use of ICS/LABA in COPD patients was associated with a higher risk of pneumonia than LAMA monotherapy. Particularly, the risk of pneumonia was high when fluticasone was used as ICS. Chronic respiratory comorbidities, a history of previous pneumonia and GOLD lower-risk group, were also at high risk for pneumonia. Thus, it is recommended that ICS use be avoided in COPD patients with high pneumonia risk.

## Supplementary Information

Supplementary Information.

## Data Availability

The datasets generated during the current study are available from the corresponding author upon reasonable request.

## Abbreviations

ASD: Absolute standardized difference
CI: Confidence interval
COPD: Chronic obstructive pulmonary disease
FDC: Fixed dose combination
GOLD: Global Initiative for Chronic Obstructive Lung Disease
HIV: Human immunodeficiency virus
HR: Hazard ratio
ICD-10: International Classification of Disease-10
ICS: Inhaled corticosteroid
LABA: Long-acting β_2_-agonist
LAMA: Long-acting muscarinic antagonist
mCCI: Modified Charlson Comorbidity Index
NHI: National Health Index
NHID: National Health Information Database
NHIS: National Health Insurance Service
PS: Propensity score
PYs: Person-years
SD: Standard deviation

## References

[1] Vogelmeier CF (2017). Global strategy for the diagnosis, management, and prevention of chronic obstructive lung disease 2017 report. GOLD executive summary. Am. J. Respir. Crit. Care Med..

[2] Lozano R (2012). Global and regional mortality from 235 causes of death for 20 age groups in 1990 and 2010: A systematic analysis for the Global Burden of Disease Study 2010. Lancet.

[3] Mannino DM, Buist AS (2007). Global burden of COPD: Risk factors, prevalence, and future trends. Lancet.

[4] Anzueto A, Miravitlles M (2018). The role of fixed-dose dual bronchodilator therapy in treating COPD. Am. J. Med..

[5] Vedel-Krogh S, Nielsen FS, Lange P, Vestbo J, Nordestgaard BG (2016). Blood eosinophils and exacerbations in chronic obstructive pulmonary disease: The Copenhagen general population study. Am. J. Respir. Crit. Care Med..

[6] Vedel-Krogh S, Nordestgaard BG, Lange P, Vestbo J, Nielsen SF (2018). Blood eosinophil count and risk of pneumonia hospitalisations in individuals with COPD. Eur. Respir. J..

[7] Singh D (2019). Global strategy for the diagnosis, management, and prevention of chronic obstructive lung disease: The GOLD science committee report 2019. Eur. Respir. J..

[8] Lee CM (2019). Inhaled corticosteroid-related tuberculosis in the real world among patients with asthma and COPD: A 10-year nationwide population-based study. J. Allergy Clin. Immunol. Pract..

[9] Yang IA, Clarke MS, Sim EH, Fong KM (2012). Inhaled corticosteroids for stable chronic obstructive pulmonary disease. Cochrane Database Syst. Rev..

[10] Crim C (2009). Pneumonia risk in COPD patients receiving inhaled corticosteroids alone or in combination: TORCH study results. Eur. Respir. J..

[11] Tashkin DP, Strange C (2018). Inhaled corticosteroids for chronic obstructive pulmonary disease: What is their role in therapy?. Int. J. Chron. Obstruct. Pulmon. Dis..

[12] Burgel PR (2014). Real-life use of inhaled corticosteroids in COPD patients versus the GOLD proposals: A paradigm shift in GOLD 2011?. Eur. Respir. J..

[13] Park JH (2019). Risk for pneumonia requiring hospitalization or emergency room visit according to delivery device for inhaled corticosteroid/long-acting beta-agonist in patients with chronic airway diseases as real-world evidence. Sci. Rep..

[14] Calverley PMA (2011). Reported pneumonia in patients with COPD: Findings from the INSPIRE study. Chest.

[15] Tashkin DP (2018). Concomitant inhaled corticosteroid use and the risk of pneumonia in COPD: A matched-subgroup post hoc analysis of the UPLIFT(R) trial. Respir. Res..

[16] Janson C (2013). Pneumonia and pneumonia related mortality in patients with COPD treated with fixed combinations of inhaled corticosteroid and long acting beta2 agonist: Observational matched cohort study (PATHOS). BMJ.

[17] Yang M, Du Y, Chen H, Jiang D, Xu Z (2019). Inhaled corticosteroids and risk of pneumonia in patients with chronic obstructive pulmonary disease: A meta-analysis of randomized controlled trials. Int. Immunopharmacol..

[18] Suissa S, Dell'Aniello S, Ernst P (2018). Comparative effectiveness of LABA-ICS versus LAMA as initial treatment in COPD targeted by blood eosinophils: A population-based cohort study. Lancet Respir. Med..

[19] Suissa S, Dell'Aniello S, Ernst P (2019). Comparative effectiveness and safety of LABA-LAMA vs LABA-ICS treatment of COPD in real-world clinical practice. Chest.

[20] Lee JH (2020). Risk of pneumonia associated with inhaled corticosteroid in patients with chronic obstructive pulmonary disease: A Korean Population-Based Study. Int. J. Chron. Obstruct. Pulmon. Dis..

[21] Lipson DA (2018). Once-daily single-inhaler triple versus dual therapy in patients with COPD. N. Engl. J. Med..

[22] Janson C, Stratelis G, Miller-Larsson A, Harrison TW, Larsson K (2017). Scientific rationale for the possible inhaled corticosteroid intraclass difference in the risk of pneumonia in COPD. Int. J. Chron. Obstruct. Pulmon. Dis..

