# Supplementary material for: Comparison of pneumonia incidence between long-acting muscarinic antagonist and inhaled corticosteroid plus long-acting beta agonist in patients with COPD
Source: Sci Rep. 2023 May 20;13:8183. doi: 10.1038/s41598-023-35223-3 (PMC10199945; doi:10.1038/s41598-023-35223-3)
Supplement: Supplementary file 1 — Supplementary Information. [file 41598_2023_35223_MOESM1_ESM.docx]

**Tables**

**Table S1**. Baseline characteristics of enrolled patients

| **Characteristics** | Unmatched population | | | | | | | | Propensity score-matched population | | | | | | | |
| --- | --- | --- | --- | --- | --- | --- | --- | --- | --- | --- | --- | --- | --- | --- | --- | --- |
|  | All  (n=4,699) | | LAMA  (n=3,479) | | ICS/LABA  (n=1,220) | | p-value | ASD | All  (n=2,006) | | LAMA  (n=1,003) | | ICS/LABA  (n=1,003) | | p-value | ASD |
|  | n | % | n | % | n | % |  |  | n | % | n | % | n | % |  |  |
| Observation period (days) |  |  |  |  |  |  |  |  |  |  |  |  |  |  |  |  |
| Mean±SD | 700.04 | 574.80 | 725.4 | 590.93 | 627.72 | 519.56 | 0.00 |  | 677.25 | 585.75 | 750.23 | 670.21 | 604.27 | 476.13 | 0.00 |  |
| Age (years) |  |  |  |  |  |  |  |  |  |  |  |  |  |  |  |  |
| Mean±SD | 69.63 | 7.91 | 69.62 | 7.85 | 69.67 | 8.08 | 0.87 | 0.01 | 69.57 | 7.97 | 69.73 | 7.77 | 69.4 | 8.17 | 0.29 | 0.04 |
| Median | 69 |  | 69 |  | 69 |  |  |  | 69 |  | 70 |  | 69 |  |  |  |
| Min | 55 |  | 55 |  | 55 |  |  |  | 55 |  | 55 |  | 55 |  |  |  |
| Max | 102 |  | 102 |  | 94 |  |  |  | 98 |  | 98 |  | 94 |  |  |  |
| P25 | 64 |  | 64 |  | 64 |  |  |  | 64 |  | 64 |  | 63 |  |  |  |
| P75 | 75 |  | 75 |  | 76 |  |  |  | 75 |  | 75 |  | 75 |  |  |  |
| 55 to < 75 | 3375 | 0.72 | 2513 | 0.72 | 862 | 0.71 | 0.29 | 0.05 | 1424 | 0.71 | 711 | 0.71 | 713 | 0.71 | 0.92 | 0.08 |
| 75+ | 1324 | 0.28 | 966 | 0.28 | 358 | 0.29 |  |  | 582 | 0.29 | 292 | 0.29 | 290 | 0.29 |  |  |
| Sex |  |  |  |  |  |  |  |  |  |  |  |  |  |  |  |  |
| Male | 3889 | 0.83 | 2991 | 0.86 | 898 | 0.74 | 0.00 | 0.31 | 1531 | 0.76 | 771 | 0.77 | 760 | 0.76 | 0.56 | 0.03 |
| Female | 810 | 0.17 | 488 | 0.14 | 322 | 0.26 |  |  | 475 | 0.24 | 232 | 0.23 | 243 | 0.24 |  |  |
| Income level |  |  |  |  |  |  |  |  |  |  |  |  |  |  |  |  |
| 1st quartile | 686 | 0.15 | 513 | 0.15 | 173 | 0.14 | 0.00 | 0.41 | 326 | 0.16 | 167 | 0.17 | 159 | 0.16 | 0.97 | 0.03 |
| 2nd quartile | 651 | 0.14 | 509 | 0.15 | 142 | 0.12 |  |  | 254 | 0.13 | 125 | 0.12 | 129 | 0.13 |  |  |
| 3rd quartile | 928 | 0.20 | 733 | 0.21 | 195 | 0.16 |  |  | 347 | 0.17 | 176 | 0.18 | 171 | 0.17 |  |  |
| 4th quartile | 1653 | 0.35 | 1290 | 0.37 | 363 | 0.30 |  |  | 677 | 0.34 | 338 | 0.34 | 339 | 0.34 |  |  |
| Medical aid | 781 | 0.17 | 434 | 0.12 | 347 | 0.28 |  |  | 402 | 0.20 | 197 | 0.20 | 205 | 0.20 |  |  |
| Hospital type |  |  |  |  |  |  |  |  |  |  |  |  |  |  |  |  |
| General hospital | 4036 | 0.86 | 3024 | 0.87 | 1012 | 0.83 | 0.00 | 0.15 | 1707 | 0.85 | 860 | 0.86 | 847 | 0.84 | 0.78 | 0.05 |
| Hospital | 251 | 0.05 | 174 | 0.05 | 77 | 0.06 |  |  | 134 | 0.07 | 62 | 0.06 | 72 | 0.07 |  |  |
| Clinic | 394 | 0.08 | 276 | 0.08 | 118 | 0.10 |  |  | 158 | 0.08 | 77 | 0.08 | 81 | 0.08 |  |  |
| Others | 18 | 0.00 | 5 | 0.00 | 13 | 0.01 |  |  | 7 | 0.00 | 4 | 0.00 | 3 | 0.00 |  |  |
| History of COPD exacerbation |  |  |  |  |  |  |  |  |  |  |  |  |  |  |  |  |
| None | 3956 | 0.84 | 2930 | 0.84 | 1026 | 0.84 | 0.91 | 0.01 | 1651 | 0.82 | 824 | 0.82 | 827 | 0.82 | 0.66 | 0.04 |
| 1 moderate | 320 | 0.07 | 239 | 0.07 | 81 | 0.07 |  |  | 145 | 0.07 | 69 | 0.07 | 76 | 0.08 |  |  |
| ≥ 2 moderate OR ≥ 1 severe | 423 | 0.09 | 310 | 0.09 | 113 | 0.09 |  |  | 210 | 0.10 | 110 | 0.11 | 100 | 0.10 |  |  |
| History of pneumonia |  |  |  |  |  |  |  |  |  |  |  |  |  |  |  |  |
| No | 4238 | 0.90 | 3113 | 0.89 | 1125 | 0.92 | 0.01 | 0.09 | 1831 | 0.91 | 917 | 0.91 | 914 | 0.91 | 0.81 | 0.01 |
| Yes | 461 | 0.10 | 366 | 0.11 | 95 | 0.08 |  |  | 175 | 0.09 | 86 | 0.09 | 89 | 0.09 |  |  |
| mCCI |  |  |  |  |  |  |  |  |  |  |  |  |  |  |  |  |
| Mean±SD | 1.81 | 1.94 | 1.85 | 1.93 | 1.67 | 1.96 | 0.00 | 0.09 | 1.87 | 2.05 | 1.87 | 2.12 | 1.87 | 1.99 | 0.61 | 0.00 |
| Median | 1 |  |  |  |  |  |  |  | 1 |  | 1 |  | 1 |  |  |  |
| Min | 0 |  |  |  |  |  |  |  | 0 |  | 0 |  | 0 |  |  |  |
| Max | 15 |  |  |  |  |  |  |  | 15 |  | 15 |  | 13 |  |  |  |
| P25 | 0 |  |  |  |  |  |  |  | 0 |  | 0 |  | 0 |  |  |  |
| P75 | 3 |  |  |  |  |  |  |  | 3 |  | 3 |  | 3 |  |  |  |
| 0,1 | 2694 | 0.57 | 1946 | 0.56 | 748 | 0.61 | 0.00 | 0.12 | 1150 | 0.57 | 582 | 0.58 | 568 | 0.57 | 0.79 | 0.04 |
| 2 | 567 | 0.12 | 436 | 0.13 | 131 | 0.11 |  |  | 236 | 0.12 | 113 | 0.11 | 123 | 0.12 |  |  |
| 3 | 780 | 0.17 | 608 | 0.17 | 172 | 0.14 |  |  | 312 | 0.16 | 159 | 0.16 | 153 | 0.15 |  |  |
| 4+ | 658 | 0.14 | 489 | 0.14 | 169 | 0.14 |  |  | 308 | 0.15 | 149 | 0.15 | 159 | 0.16 |  |  |
| mCCI category |  |  |  |  |  |  |  |  |  |  |  |  |  |  |  |  |
| Congestive heart failure | 459 | 0.10 | 345 | 0.10 | 114 | 0.09 | 0.56 | 0.02 | 207 | 0.10 | 99 | 0.10 | 108 | 0.11 | 0.51 | 0.03 |
| Dementia | 165 | 0.04 | 128 | 0.04 | 37 | 0.03 | 0.29 | 0.04 | 70 | 0.03 | 33 | 0.03 | 37 | 0.04 | 0.63 | 0.02 |
| Chronic pulmonary disease | 2613 | 0.56 | 2003 | 0.58 | 610 | 0.50 | 0.00 | 0.15 | 1117 | 0.56 | 563 | 0.56 | 554 | 0.55 | 0.69 | 0.02 |
| Rheumatologic disease | 171 | 0.04 | 115 | 0.03 | 56 | 0.05 | 0.04 | 0.07 | 103 | 0.05 | 50 | 0.05 | 53 | 0.05 | 0.76 | 0.02 |
| Mild liver disease | 979 | 0.21 | 743 | 0.21 | 236 | 0.19 | 0.14 | 0.05 | 422 | 0.21 | 205 | 0.20 | 217 | 0.22 | 0.51 | 0.03 |
| Diabetes with chronic complications | 471 | 0.10 | 354 | 0.10 | 117 | 0.10 | 0.56 | 0.02 | 203 | 0.10 | 93 | 0.09 | 110 | 0.11 | 0.21 | 0.06 |
| Hemiplegia or paraplegia | 52 | 0.01 | 41 | 0.01 | 11 | 0.01 | 0.43 | 0.03 | 20 | 0.01 | 9 | 0.01 | 11 | 0.01 | 0.65 | 0.02 |
| Renal disease | 130 | 0.03 | 91 | 0.03 | 39 | 0.03 | 0.29 | 0.03 | 76 | 0.04 | 39 | 0.04 | 37 | 0.04 | 0.82 | 0.01 |
| Any malignancy, including lymphoma and leukemia | 630 | 0.13 | 484 | 0.14 | 146 | 0.12 | 0.09 | 0.06 | 267 | 0.13 | 136 | 0.14 | 131 | 0.13 | 0.74 | 0.01 |
| Moderate or severe liver disease | 41 | 0.01 | 30 | 0.01 | 11 | 0.01 | 0.90 | 0.00 | 23 | 0.01 | 12 | 0.01 | 11 | 0.01 | 0.83 | 0.01 |
| Metastatic solid tumor | 60 | 0.01 | 46 | 0.01 | 14 | 0.01 | 0.64 | 0.02 | 32 | 0.02 | 20 | 0.02 | 12 | 0.01 | 0.15 | 0.07 |
| HIV | 1 | 0.00 | 0 | 0.00 | 1 | 0.00 | 0.09 | NA | 0 | 0.00 | 0 | 0.00 | 0 | 0.00 | NA | NA |
| Index year |  |  |  |  |  |  |  |  |  |  |  |  |  |  |  |  |
| 2005 | 361 | 0.08 | 77 | 0.02 | 285 | 0.23 | 0.00 | 0.75 | 161 | 0.08 | 77 | 0.08 | 84 | 0.08 | 0.99 | 0.07 |
| 2006 | 306 | 0.07 | 185 | 0.05 | 121 | 0.10 |  |  | 230 | 0.11 | 123 | 0.12 | 107 | 0.11 |  |  |
| 2007 | 363 | 0.08 | 258 | 0.07 | 105 | 0.09 |  |  | 212 | 0.11 | 107 | 0.11 | 105 | 0.10 |  |  |
| 2008 | 453 | 0.10 | 374 | 0.11 | 79 | 0.06 |  |  | 158 | 0.08 | 79 | 0.08 | 79 | 0.08 |  |  |
| 2009 | 490 | 0.10 | 379 | 0.11 | 111 | 0.09 |  |  | 220 | 0.11 | 110 | 0.11 | 110 | 0.11 |  |  |
| 2010 | 585 | 0.12 | 481 | 0.14 | 104 | 0.09 |  |  | 202 | 0.10 | 98 | 0.10 | 104 | 0.10 |  |  |
| 2011 | 566 | 0.12 | 461 | 0.13 | 105 | 0.09 |  |  | 214 | 0.11 | 110 | 0.11 | 104 | 0.10 |  |  |
| 2012 | 545 | 0.12 | 441 | 0.13 | 104 | 0.09 |  |  | 208 | 0.10 | 104 | 0.10 | 104 | 0.10 |  |  |
| 2013 | 447 | 0.10 | 363 | 0.10 | 84 | 0.07 |  |  | 159 | 0.08 | 75 | 0.07 | 84 | 0.08 |  |  |
| 2014 | 465 | 0.10 | 369 | 0.11 | 96 | 0.08 |  |  | 191 | 0.10 | 95 | 0.09 | 96 | 0.10 |  |  |
| 2015 | 117 | 0.02 | 91 | 0.03 | 26 | 0.02 |  |  | 51 | 0.03 | 25 | 0.02 | 26 | 0.03 |  |  |

Abbreviations: Long-acting muscarinic antagonists, LAMA; Inhaled corticosteroid, ICS; Long-acting β2-agonists, LABA; Absolute standardized difference, ASD; Standard deviation, SD; Chronic obstructive pulmonary disease, COPD; Modified Charlson comorbidity index, mCCI; Human immunodeficiency virus, HIV

**Table S2**. Incidence rate of pneumonia

| **Pneumonia** | Unmatched population | | | | Propensity score-matched population | | | |
| --- | --- | --- | --- | --- | --- | --- | --- | --- |
|  | All | LAMA | ICS/LABA | p-value | All | LAMA | ICS/LABA | p-value |
| Incidence rate per 1,000 PYs | 102.78 | 90.20 | 147.09 | <.0001 | 112.41 | 93.96 | 136.42 | 0.0004 |
| Patients with event | 812 | 555 | 257 |  | 366 | 173 | 193 |  |
| Sum of PYs | 7900.29 | 6153.06 | 1747.23 |  | 3256.03 | 1841.3 | 1414.73 |  |
| Time to event |  |  |  |  |  |  |  |  |
| Mean | 432.61 | 446.42 | 402.79 |  | 441.61 | 523.64 | 368.08 |  |
| SD | 548.35 | 563.61 | 513.63 |  | 568.5 | 675.18 | 441.04 |  |
| Median | 257.5 | 256 | 265 |  | 265.5 | 280 | 259 |  |
| Min | 2 | 2 | 2 |  | 2 | 2 | 2 |  |
| Max | 3481 | 3481 | 2995 |  | 3481 | 3481 | 2791 |  |
| P25 | 96 | 96 | 93 |  | 103 | 112 | 98 |  |
| P75 | 495 | 525 | 471 |  | 514 | 604 | 437 |  |
| Age |  |  |  |  |  |  |  |  |
| 55 to <75 | 90.35 | 77.90 | 136.62 | <.0001 | 100.45 | 82.37 | 125.05 | 0.0013 |
| 75+ | 137.84 | 126.49 | 172.47 | 0.0179 | 143.86 | 126.66 | 163.82 | 0.1443 |
| Sex |  |  |  |  |  |  |  |  |
| Male | 100.99 | 89.96 | 146.01 | <.0001 | 114.73 | 97.85 | 136.73 | 0.0046 |
| Female | 111.66 | 91.67 | 150.17 | 0.0031 | 104.59 | 80.80 | 135.40 | 0.0230 |
| History of COPD exacerbation |  |  |  |  |  |  |  |  |
| None | 95.27 | 82.78 | 139.34 | <.0001 | 105.25 | 88.78 | 126.5 | 0.0029 |
| 1 moderate | 140.57 | 122.65 | 214.09 | 0.0453 | 142.28 | 84.91 | 220.8 | 0.0116 |
| ≥ 2 moderate OR ≥ 1 severe | 149.54 | 140.35 | 178.36 | 0.2767 | 149.82 | 137.98 | 165.74 | 0.5064 |

Abbreviations: long-acting muscarinic antagonists, LAMA; inhaled corticosteroid, ICS; long-acting β2-agonists, LABA; Person years, PYs; Standard deviation, SD; Chronic obstructive pulmonary disease, COPD

**Table S3**. Incidence rate of pneumonia-related hospitalization

| **Pneumonia-related hospitalization** | Unmatched population | | | | Propensity score-matched population | | | |
| --- | --- | --- | --- | --- | --- | --- | --- | --- |
|  | All | LAMA | ICS/LABA | p-value | All | LAMA | ICS/LABA | p-value |
| Incidence rate per 1,000 PYs | 59.88 | 49.57 | 95.87 | <.0001 | 67.56 | 52.55 | 86.89 | 0.0001 |
| Patients with event | 502 | 323 | 179 |  | 233 | 102 | 131 |  |
| Sum of PYs | 8383.02 | 6515.88 | 1867.14 |  | 3448.58 | 1940.98 | 1507.59 |  |
| Time to event |  |  |  |  |  |  |  |  |
| Mean | 495.86 | 519.22 | 453.72 |  | 497.45 | 572.75 | 438.82 |  |
| SD | 595.92 | 622.08 | 544.7 |  | 624 | 743.3 | 507.68 |  |
| Median | 295.5 | 298 | 295 |  | 301 | 308.5 | 296 |  |
| Min | 2 | 2 | 2 |  | 2 | 2 | 2 |  |
| Max | 3481 | 3481 | 2791 |  | 3481 | 3481 | 2791 |  |
| P25 | 116 | 120 | 109 |  | 130 | 141 | 115 |  |
| P75 | 602 | 681 | 574 |  | 595 | 636 | 595 |  |
| Age |  |  |  |  |  |  |  |  |
| 55 to <75 | 48.51 | 39.23 | 82.36 | <.0001 | 56.21 | 43.93 | 72.61 | 0.0029 |
| 75+ | 92.27 | 80.16 | 129.8 | 0.0016 | 97.85 | 76.95 | 122.29 | 0.0270 |
| Sex |  |  |  |  |  |  |  |  |
| Male | 59.48 | 50.00 | 97.87 | <.0001 | 71.53 | 55.97 | 91.74 | 0.0007 |
| Female | 61.83 | 47.03 | 90.21 | 0.0025 | 54.27 | 40.90 | 70.98 | 0.0721 |
| History of COPD exacerbation |  |  |  |  |  |  |  |  |
| None | 55.98 | 45.30 | 93.47 | <.0001 | 64.02 | 49.48 | 82.65 | 0.0006 |
| 1 moderate | 76.75 | 65.08 | 122.31 | 0.0733 | 75.30 | 39.42 | 121.31 | 0.0258 |
| ≥ 2 moderate OR ≥ 1 severe | 84.42 | 79.08 | 100.94 | 0.3791 | 89.49 | 82.79 | 98.45 | 0.6148 |

Abbreviations: long-acting muscarinic antagonists, LAMA; inhaled corticosteroid, ICS; long-acting β2-agonists, LABA; Person years, PYs; Standard deviation, SD; Chronic obstructive pulmonary disease, COPD

**Table S4**. Incidence rate of outpatient pneumonia events

| **Outpatient pneumonia events** | Unmatched population | | | | Propensity score-matched population | | | |
| --- | --- | --- | --- | --- | --- | --- | --- | --- |
|  | All | LAMA | ICS/LABA | p-value | All | LAMA | ICS/LABA | p-value |
| Incidence rate per 1,000 PYs | 52.89 | 49.14 | 65.58 | 0.0075 | 54.33 | 47.55 | 62.93 | 0.0549 |
| Patients with event | 442 | 317 | 125 |  | 188 | 92 | 96 |  |
| Sum of PYs | 8356.53 | 6450.45 | 1906.08 |  | 3460.45 | 1934.86 | 1525.59 |  |
| Time to event |  |  |  |  |  |  |  |  |
| Mean | 649.55 | 677.21 | 570.65 |  | 630.07 | 704.6 | 555.55 |  |
| SD | 550.5 | 570.73 | 479.75 |  | 564.09 | 645.38 | 457.26 |  |
| Median | 366 | 366 | 365 |  | 365 | 366 | 365 |  |
| Min | 4 | 7 | 4 |  | 4 | 7 | 4 |  |
| Max | 4009 | 4009 | 3694 |  | 4009 | 4009 | 3694 |  |
| P25 | 365 | 365 | 365 |  | 365 | 365 | 365 |  |
| P75 | 731 | 731 | 730 |  | 730 | 731 | 730 |  |
| Age |  |  |  |  |  |  |  |  |
| 55 to <75 | 51.19 | 47.12 | 65.70 | 0.0100 | 53.52 | 45.11 | 64.71 | 0.0377 |
| 75+ | 57.53 | 54.90 | 65.30 | 0.3790 | 56.39 | 54.26 | 58.78 | 0.7666 |
| Sex |  |  |  |  |  |  |  |  |
| Male | 50.62 | 47.65 | 62.29 | 0.0334 | 52.72 | 47.43 | 59.45 | 0.1805 |
| Female | 64.15 | 58.27 | 75.00 | 0.2427 | 59.78 | 47.94 | 74.67 | 0.1295 |
| History of COPD exacerbation |  |  |  |  |  |  |  |  |
| None | 48.80 | 45.21 | 60.87 | 0.0144 | 51.03 | 46.57 | 56.61 | 0.2389 |
| 1 moderate | 77.27 | 67.00 | 117.95 | 0.1175 | 78.99 | 41.28 | 127.53 | 0.0252 |
| ≥ 2 moderate OR ≥ 1 severe | 74.84 | 73.68 | 78.66 | 0.8326 | 65.08 | 57.87 | 75.23 | 0.5137 |

Abbreviations: long-acting muscarinic antagonists, LAMA; inhaled corticosteroid, ICS; long-acting β2-agonists, LABA; Person years, PYs; Standard deviation, SD; Chronic obstructive pulmonary disease, COPD

**Table S5**. Incidence rate of pneumonia-related death

| **Pneumonia-related death** | Unmatched population | | | | Propensity score-matched population | | | |
| --- | --- | --- | --- | --- | --- | --- | --- | --- |
|  | All | LAMA | ICS/LABA | p-value | All | LAMA | ICS/LABA | p-value |
| Incidence rate per 1,000 PYs | 2.55 | 2.17 | 3.82 | 0.2130 | 2.96 | 1.94 | 4.22 | 0.2050 |
| Patients with event | 23 | 15 | 8 |  | 11 | 4 | 7 |  |
| Sum of PYs | 9006.12 | 6909.43 | 2096.69 |  | 3719.53 | 2060.18 | 1659.35 |  |
| Time to event |  |  |  |  |  |  |  |  |
| Mean | 765.17 | 659.07 | 964.13 |  | 894.09 | 771.75 | 964 |  |
| SD | 753.38 | 657.7 | 921.58 |  | 934.95 | 949.06 | 995.42 |  |
| Median | 361 | 353 | 620.5 |  | 361 | 357 | 535 |  |
| Min | 93 | 93 | 163 |  | 163 | 183 | 163 |  |
| Max | 2846 | 2190 | 2846 |  | 2846 | 2190 | 2846 |  |
| P25 | 329 | 276 | 339.5 |  | 336 | 268 | 336 |  |
| P75 | 965 | 728 | 1392 |  | 1819 | 1275.5 | 1819 |  |
| Age |  |  |  |  |  |  |  |  |
| 55 to <75 | 1.82 | 1.37 | 3.37 | 0.1396 | 2.61 | 1.99 | 3.43 | 0.4717 |
| 75+ | 4.56 | 4.45 | 4.90 | 0.8873 | 3.84 | 1.82 | 6.09 | 0.2583 |
| Sex |  |  |  |  |  |  |  |  |
| Male | 2.55 | 2.20 | 3.88 | 0.2685 | 3.48 | 2.50 | 4.71 | 0.3205 |
| Female | 2.58 | 2.00 | 3.63 | 0.5554 | 1.18 | 0.00 | 2.59 | 0.2098 |
| History of COPD exacerbation |  |  |  |  |  |  |  |  |
| None | 2.11 | 1.72 | 3.39 | 0.2056 | 2.62 | 1.19 | 4.38 | 0.0828 |
| 1 moderate | 8.91 | 9.09 | 8.26 | 0.9314 | 4.11 | 7.68 | 0.00 | 0.2630 |
| ≥ 2 moderate OR ≥ 1 severe | 2.34 | 1.53 | 4.92 | 0.4222 | 4.75 | 4.09 | 5.67 | 0.8183 |

Abbreviations: long-acting muscarinic antagonists, LAMA; inhaled corticosteroid, ICS; long-acting β2-agonists, LABA; Person years, PYs; Standard deviation, SD; Chronic obstructive pulmonary disease, COPD

**Table S6**. Frequency of pneumonia events

| **Pneumonia events** | Unmatched population | | | | Propensity score-matched population | | | |
| --- | --- | --- | --- | --- | --- | --- | --- | --- |
|  | All | LAMA | ICS/LABA | p-value | All | LAMA | ICS/LABA | p-value |
| Number of events per 1,000 persons per year | 170.73 | 153.12 | 220.94 | <.001 | 176.75 | 155.16 | 198.35 | 0.172 |
| Total events | 1358 | 902 | 456 |  | 575 | 250 | 325 |  |
| Patients with event | 812 | 555 | 257 |  | 366 | 173 | 193 |  |
| Follow-up period (years) |  |  |  |  |  |  |  |  |
| Mean | 1.92 | 1.99 | 1.72 |  | 1.85 | 2.05 | 1.65 |  |
| SD | 1.57 | 1.62 | 1.42 |  | 1.6 | 1.83 | 1.3 |  |
| Median | 1 | 1 | 1 |  | 1 | 1 | 1 |  |
| Min | 0.25 | 0.25 | 0.45 |  | 0.45 | 0.5 | 0.45 |  |
| Max | 10.98 | 10.98 | 10.34 |  | 10.98 | 10.98 | 10.11 |  |
| P25 | 1 | 1 | 1 |  | 1 | 1 | 1 |  |
| P75 | 2 | 2 | 2 |  | 2 | 2 | 2 |  |
| Age |  |  |  |  |  |  |  |  |
| 55 to <75 | 156.24 | 138.31 | 208.49 | 0.000 | 162.56 | 145.07 | 180.01 | 0.268 |
| 75+ | 207.67 | 191.64 | 250.93 | 0.092 | 211.47 | 179.72 | 243.44 | 0.434 |
| Sex |  |  |  |  |  |  |  |  |
| Male | 167.47 | 153.39 | 214.39 | <.001 | 178.46 | 162.51 | 194.65 | 0.313 |
| Female | 186.36 | 151.48 | 239.21 | 0.079 | 171.24 | 130.72 | 209.92 | 0.292 |
| History of COPD exacerbation |  |  |  |  |  |  |  |  |
| None | 158.62 | 138.62 | 215.74 | <.001 | 167.99 | 148.07 | 187.83 | 0.285 |
| 1 moderate | 202.03 | 186.38 | 248.21 | 0.253 | 163.01 | 67.64 | 249.60 | 0.062 |
| ≥ 2 moderate OR ≥ 1 severe | 260.29 | 264.52 | 248.69 | 0.672 | 255.16 | 263.11 | 246.42 | 0.863 |

Abbreviations: long-acting muscarinic antagonists, LAMA; inhaled corticosteroid, ICS; long-acting β2-agonists, LABA; chronic obstructive pulmonary disease, COPD

**Table S7**. Frequency of pneumonia-related hospitalization events

| **Pneumonia-related hospitalization events** | Unmatched population | | | | Propensity score-matched population | | | |
| --- | --- | --- | --- | --- | --- | --- | --- | --- |
|  | All | LAMA | ICS/LABA | p-value | All | LAMA | ICS/LABA | p-value |
| Number of events per 1,000 persons per year | 92.95 | 76.53 | 139.78 | <.001 | 101.01 | 80.18 | 121.84 | 0.036 |
| Total events | 711 | 433 | 278 |  | 316 | 127 | 189 |  |
| Patients with event | 502 | 323 | 179 |  | 233 | 102 | 131 |  |
| Observation period (years) |  |  |  |  |  |  |  |  |
| Mean | 1.92 | 1.99 | 1.72 |  | 1.85 | 2.05 | 1.65 |  |
| SD | 1.57 | 1.62 | 1.42 |  | 1.6 | 1.83 | 1.3 |  |
| Median | 1 | 1 | 1 |  | 1 | 1 | 1 |  |
| Min | 0.25 | 0.25 | 0.45 |  | 0.45 | 0.5 | 0.45 |  |
| Max | 10.98 | 10.98 | 10.34 |  | 10.98 | 10.98 | 10.11 |  |
| P25 | 1 | 1 | 1 |  | 1 | 1 | 1 |  |
| P75 | 2 | 2 | 2 |  | 2 | 2 | 2 |  |
| Age |  |  |  |  |  |  |  |  |
| 55 to <75 | 76.81 | 60.92 | 123.13 | 0.000 | 83.87 | 69.00 | 99.69 | 0.165 |
| 75+ | 134.10 | 117.14 | 179.88 | 0.012 | 142.96 | 107.41 | 178.76 | 0.100 |
| Sex |  |  |  |  |  |  |  |  |
| Male | 91.23 | 77.84 | 135.83 | <.001 | 105.59 | 86.51 | 124.95 | 0.055 |
| Female | 101.21 | 68.49 | 150.81 | 0.035 | 86.26 | 59.15 | 112.11 | 0.354 |
| History of COPD exacerbation |  |  |  |  |  |  |  |  |
| None | 88.02 | 70.18 | 138.94 | <.001 | 98.86 | 79.68 | 117.98 | 0.055 |
| 1 moderate | 117.95 | 110.07 | 141.20 | 0.271 | 87.10 | 33.74 | 135.55 | 0.102 |
| ≥ 2 moderate OR ≥ 1 severe | 120.21 | 110.66 | 146.42 | 0.582 | 127.50 | 113.08 | 143.36 | 0.979 |

Abbreviations: long-acting muscarinic antagonists, LAMA; inhaled corticosteroid, ICS; long-acting β2-agonists, LABA; chronic obstructive pulmonary disease, COPD

**Table S8**. Frequency of outpatient pneumonia events

| **Outpatient pneumonia events** | Unmatched population | | | | Propensity score-matched population | | | |
| --- | --- | --- | --- | --- | --- | --- | --- | --- |
|  | All | LAMA | ICS/LABA | p-value | All | LAMA | ICS/LABA | p-value |
| Number of events per 1,000 persons per year | 77.88 | 76.73 | 81.16 | 0.240 | 75.74 | 74.98 | 76.51 | 0.738 |
| Total events | 648 | 470 | 178 |  | 259 | 123 | 136 |  |
| Patients with event | 442 | 317 | 125 |  | 188 | 92 | 96 |  |
| Observation period (years) |  |  |  |  |  |  |  |  |
| Mean | 1.92 | 1.99 | 1.72 |  | 1.85 | 2.05 | 1.65 |  |
| SD | 1.57 | 1.62 | 1.42 |  | 1.6 | 1.83 | 1.3 |  |
| Median | 1 | 1 | 1 |  | 1 | 1 | 1 |  |
| Min | 0.25 | 0.25 | 0.45 |  | 0.45 | 0.5 | 0.45 |  |
| Max | 10.98 | 10.98 | 10.34 |  | 10.98 | 10.98 | 10.11 |  |
| P25 | 1 | 1 | 1 |  | 1 | 1 | 1 |  |
| P75 | 2 | 2 | 2 |  | 2 | 2 | 2 |  |
| Age |  |  |  |  |  |  |  |  |
| 55 to <75 | 79.57 | 77.59 | 85.36 | 0.258 | 78.70 | 76.07 | 81.32 | 0.628 |
| 75+ | 73.57 | 74.50 | 71.05 | 0.691 | 68.51 | 72.31 | 64.68 | 0.878 |
| Sex |  |  |  |  |  |  |  |  |
| Male | 76.37 | 75.71 | 78.56 | 0.360 | 72.87 | 76.00 | 69.70 | 0.982 |
| Female | 85.14 | 83.00 | 88.40 | 0.793 | 85.00 | 71.57 | 97.81 | 0.537 |
| History of COPD exacerbation |  |  |  |  |  |  |  |  |
| None | 70.73 | 68.61 | 76.79 | 0.223 | 69.12 | 68.40 | 69.85 | 0.888 |
| 1 moderate | 84.08 | 76.31 | 107.01 | 0.402 | 75.91 | 33.90 | 114.05 | 0.097 |
| ≥ 2 moderate OR ≥ 1 severe | 140.08 | 153.86 | 102.27 | 0.634 | 127.66 | 150.03 | 103.06 | 0.952 |

Abbreviations: long-acting muscarinic antagonists, LAMA; inhaled corticosteroid, ICS; long-acting β2-agonists, LABA; chronic obstructive pulmonary disease, COPD

**Table S9**. Hazard ratios of pneumonia

| **Pneumonia** | Unmatched population | | | | | | | | | | Propensity score-matched population | | | | | | |
| --- | --- | --- | --- | --- | --- | --- | --- | --- | --- | --- | --- | --- | --- | --- | --- | --- | --- |
|  | Event | | No event | | Crude HR | | | Adjusted HR | | | Event | | No event | | Crude HR | | |
|  | n | % | n | % | HR | 95% CI | p-value | HR | 95% CI | p-value | n | % | n | % | HR | 95% CI | p-value |
| Overall | 812 | 0.17 | 3887 | 0.83 | 1.550 | 1.337-1.797 | 0.2068 | 1.427 | 1.209-1.684 | <.0001 | 366 | 0.18 | 1640 | 0.82 | 1.374 | 1.116-1.692 | 0.0028 |
| Age | Interaction: 0.318 | | | | | | | | | | Interaction: 0.790 | | | | | | |
| 55 to < 75 | 527 | 0.16 | 2848 | 0.84 | 1.638 | 1.363-1.969 | 0.1322 | 1.479 | 1.203-1.818 | 0.0002 | 237 | 0.17 | 1187 | 0.83 | 1.390 | 1.073-1.800 | 0.0126 |
| 75+ | 285 | 0.22 | 1039 | 0.78 | 1.322 | 1.034-1.691 | 0.0298 | 1.324 | 1.000-1.754 | 0.0500 | 129 | 0.22 | 453 | 0.78 | 1.270 | 0.901-1.791 | 0.1724 |
| Sex | Interaction: 0.841 | | | | | | | | | | Interaction: 0.520 | | | | | | |
| Male | 664 | 0.17 | 3225 | 0.83 | 1.545 | 1.306-1.829 | 0.2393 | 1.431 | 1.188-1.723 | 0.0002 | 288 | 0.19 | 1243 | 0.81 | 1.324 | 1.048-1.674 | 0.0187 |
| Female | 148 | 0.18 | 662 | 0.82 | 1.535 | 1.110-2.124 | 0.6155 | 1.445 | 0.995-2.099 | 0.0533 | 78 | 0.16 | 397 | 0.84 | 1.564 | 0.994-2.462 | 0.0531 |
| Income level | Interaction: 0.953 | | | | | | | | | | Interaction: 0.907 | | | | | | |
| 1st quartile | 102 | 0.15 | 584 | 0.85 | 1.353 | 0.879-2.083 | 0.8400 | 1.240 | 0.760-2.021 | 0.3889 | 54 | 0.17 | 272 | 0.83 | 1.224 | 0.714-2.097 | 0.4624 |
| 2nd quartile | 112 | 0.17 | 539 | 0.83 | 1.652 | 1.083-2.519 | 0.1938 | 1.629 | 1.037-2.561 | 0.0344 | 48 | 0.19 | 206 | 0.81 | 0.680 | 0.942-2.994 | 0.0787 |
| 3rd quartile | 149 | 0.16 | 779 | 0.84 | 1.333 | 0.910-1.951 | 0.7875 | 1.366 | 0.904-2.064 | 0.1385 | 62 | 0.18 | 285 | 0.82 | 1.143 | 0.687-1.900 | 0.6068 |
| 4th quartile | 272 | 0.16 | 1381 | 0.84 | 1.494 | 1.135-1.966 | 0.0867 | 1.426 | 1.061-1.915 | 0.0185 | 114 | 0.17 | 563 | 0.83 | 1.474 | 1.011-2.150 | 0.0440 |
| Medical aid | 177 | 0.23 | 604 | 0.77 | 1.566 | 1.166-2.103 | 0.6969 | 1.279 | 0.891-1.835 | 0.1824 | 88 | 0.22 | 314 | 0.78 | 1.386 | 0.908-2.117 | 0.1308 |
| Hospital type | Interaction: 0.901 | | | | | | | | | | Interaction: 0.893 | | | | | | |
| General hospital | 709 | 0.18 | 3327 | 0.82 | 1563 | 1.333-1.833 | 0.3272 | 1.420 | 1.187-1.699 | 0.0001 | 322 | 0.19 | 1385 | 0.81 | 1.327 | 1.064-1.656 | 0.0122 |
| Hospital | 42 | 0.17 | 209 | 0.83 | NA | NA | NA | 1.366 | 0.641-2.908 | 0.4190 | 18 | 0.13 | 116 | 0.87 | 2.530 | 0.885-7.235 | 0.0834 |
| Clinic | 59 | 0.15 | 335 | 0.85 | 2.075 | 1.228-3.506 | <.0001 | 1.145 | 0.571-2.297 | 0.7021 | 25 | 0.16 | 133 | 0.84 | 1.516 | 0.678-3.391 | 0.3107 |
| Others | 2 | 0.11 | 16 | 0.89 | NA | NA | NA | NA | NA | NA | 1 | 0.14 | 6 | 0.86 | NA | NA | NA |
| History of COPD exacerbation | Interaction: 0.867 | | | | | | | | | | Interaction: 0.286 | | | | | | |
| None | 641 | 0.16 | 3315 | 0.84 | 1.605 | 1.359-1.894 | 0.2191 | 1.406 | 1.162-1.700 | 0.0004 | 284 | 0.17 | 1367 | 0.83 | 1.340 | 1.058-1.696 | 0.0150 |
| 1 moderate | 67 | 0.21 | 253 | 0.79 | 1.743 | 1.028-2.955 | 0.7990 | 2.030 | 1.092-3.771 | 0.0252 | 29 | 0.20 | 116 | 0.80 | 3.446 | 1.452-8.182 | 0.0050 |
| ≥ 2 moderate OR ≥ 1 severe | 104 | 0.25 | 319 | 0.75 | 1.173 | 0.780-1.765 | 0.5234 | 1.440 | 0.915-2.267 | 0.1152 | 53 | 0.25 | 157 | 0.75 | 1.089 | 0.633-1.874 | 0.7576 |
| History of pneumonia | Interaction: 0.028 | | | | | | | | | | Interaction: 0.304 | | | | | | |
| No | 664 | 0.16 | 3574 | 0.84 | 1.769 | 1.507-2.076 | 0.2431 | 1.529 | 1.277-1.831 | <.0001 | 314 | 0.17 | 1517 | 0.83 | 1.459 | 1.165-1.827 | 0.0010 |
| Yes | 148 | 0.32 | 313 | 0.68 | 0.877 | 0.577-1.333 | 0.7315 | 0.820 | 0.521-1.293 | 0.3934 | 52 | 0.30 | 123 | 0.70 | 0.918 | 0.529-1.592 | 0.7598 |
| mCCI | Interaction: 0.716 | | | | | | | | | | Interaction: 0.849 | | | | | | |
| 0,1 | 464 | 0.17 | 2230 | 0.83 | 1.599 | 1.321-1.936 | 0.5761 | 1.386 | 1.108-1.733 | 0.0043 | 207 | 0.18 | 943 | 0.82 | 1.309 | 0.993-1.725 | 0.0557 |
| 2 | 77 | 0.14 | 490 | 0.86 | 1.471 | 0.880-2.460 | 0.9146 | 1.792 | 1.030-3.118 | 0.0391 | 38 | 0.16 | 198 | 0.84 | 1.167 | 0.613-2.221 | 0.6387 |
| 3 | 142 | 0.18 | 638 | 0.82 | 1.618 | 1.122-2.335 | 0.1360 | 1.651 | 1.105-2.465 | 0.0143 | 63 | 0.20 | 249 | 0.80 | 1.377 | 0.836-2.269 | 0.2093 |
| 4+ | 129 | 0.20 | 529 | 0.80 | 1.271 | 0.868-1.860 | 0.4386 | 1.368 | 0.911-2.055 | 0.1312 | 58 | 0.19 | 250 | 0.81 | 1.639 | 0.964-2.786 | 0.0679 |
| mCCI category |  | | | | | | | | | |  | | | | | | |
| Congestive heart failure | Interaction: 0.266 | | | | | | | | | | Interaction: 0.182 | | | | | | |
| No | 729 | 0.17 | 3511 | 0.83 | 1.509 | 1.291-1.764 | <.0001 | 1.373 | 1.151-1.637 | 0.0004 | 331 | 0.18 | 1468 | 0.82 | 1.308 | 1.052-1.628 | 0.0158 |
| Yes | 83 | 0.18 | 376 | 0.82 | 1.975 | 1.258-3.102 | 0.0038 | 1.832 | 1.080-3.107 | 0.0248 | 35 | 0.17 | 172 | 0.83 | 2.277 | 1.104-4.698 | 0.0259 |
| Dementia | Interaction: 0.557 | | | | | | | | | | Interaction: 0.746 | | | | | | |
| No | 782 | 0.17 | 3752 | 0.83 | 1.568 | 1.349-1.823 | <.0001 | 1.435 | 1.212-1.699 | <.0001 | 353 | 0.18 | 1583 | 0.82 | 1.384 | 1.119-1.710 | 0.0027 |
| Yes | 30 | 0.18 | 135 | 0.82 | 1.095 | 0.469-2.557 | 0.8333 | 2.322 | 0.803-6.712 | 0.1198 | 13 | 0.19 | 57 | 0.81 | 1.148 | 0.385-3.425 | 0.8041 |
| Chronic pulmonary disease | Interaction 0.941 | | | | | | | | | | Interaction: 0.512 | | | | | | |
| No | 327 | 0.16 | 1759 | 0.84 | 1.680 | 1.345-2.100 | <.0001 | 1.326 | 1.011-1.740 | 0.0415 | 151 | 0.17 | 738 | 0.83 | 1.227 | 0.890-1.691 | 0.2124 |
| Yes | 485 | 0.19 | 2128 | 0.81 | 1.523 | 1.247-1.859 | <.0001 | 1.495 | 1.208-1.851 | 0.0002 | 215 | 0.19 | 902 | 0.81 | 1.569 | 1.189-2.069 | 0.0014 |
| Rheumatologic disease | Interaction: 0.852 | | | | | | | | | | Interaction: 0.237 | | | | | | |
| No | 777 | 0.17 | 3751 | 0.83 | 1.558 | 1.339-1.813 | <.0001 | 1.413 | 1.192-1.676 | <.0001 | 349 | 0.18 | 1554 | 0.82 | 1.341 | 1.084-1.658 | 0.0069 |
| Yes | 35 | 0.20 | 136 | 0.80 | 1.270 | 0.644-2.506 | 0.5056 | 1.010 | 0.432-2.359 | 0.9820 | 17 | 0.17 | 86 | 0.83 | 2.184 | 0.796-5.994 | 0.1294 |
| Mild liver disease | Interaction: 0.396 | | | | | | | | | | Interaction: 0.781 | | | | | | |
| No | 637 | 0.17 | 3083 | 0.83 | 1.622 | 1.376-1.912 | <.0001 | 1.481 | 1.226-1.789 | <.0001 | 285 | 0.18 | 1299 | 0.82 | 1.387 | 1.095-1.755 | 0.0066 |
| Yes | 175 | 0.18 | 804 | 0.82 | 1.310 | 0.932-1.840 | 0.1188 | 1.301 | 0.910-1.858 | 0.1489 | 81 | 0.19 | 341 | 0.81 | 1.343 | 0.862-2.092 | 0.1928 |
| Diabetes with chronic complications | Interaction: 0.819 | | | | | | | | | | Interaction: 0.307 | | | | | | |
| No | 730 | 0.17 | 3498 | 0.83 | 1.563 | 1.338-1.825 | <.0001 | 1.405 | 1.178-1.675 | 0.0002 | 331 | 0.18 | 1472 | 0.82 | 1.325 | 1.064-1.649 | 0.0117 |
| Yes | 82 | 0.17 | 389 | 0.83 | 1.451 | 0.895-2.354 | 0.1271 | 1.792 | 1.052-3.052 | 0.0317 | 35 | 0.17 | 168 | 0.83 | 2.028 | 0.999-4.115 | 0.0502 |
| Hemiplegia or paraplegia | Interaction: 0.790 | | | | | | | | | | Interaction: 0.788 | | | | | | |
| No | 803 | 0.17 | 3844 | 0.83 | 1.553 | 1.339-1.801 | <.0001 | 1.431 | 1.211-1.690 | <.0001 | 362 | 0.18 | 1624 | 0.82 | 1.378 | 1.118-1.698 | 0.0027 |
| Yes | 9 | 0.17 | 43 | 0.83 | 1.311 | 0.272-6.320 | 0.7405 | NA | NA | NA | 4 | 0.20 | 16 | 0.80 | 0.876 | 0.123-6.231 | 0.8948 |
| Renal disease | Interaction: 0.791 | | | | | | | | | | Interaction: 0.759 | | | | | | |
| No | 792 | 0.17 | 3777 | 0.83 | 1.565 | 1.347-1.817 | <.0001 | 1.432 | 1.210-1.694 | <.0001 | 354 | 0.18 | 1576 | 0.82 | 1.380 | 1.117-1.705 | 0.0028 |
| Yes | 20 | 0.15 | 110 | 0.85 | 1.127 | 0.437-2.909 | 0.8111 | 0.661 | 0.154-2.839 | 0.5779 | 12 | 0.16 | 64 | 0.84 | 1.199 | 0.366-3.929 | 0.7650 |
| Any malignancy, including lymphoma and leukemia | Interaction: 0.538 | | | | | | | | | | Interaction: 0.509 | | | | | | |
| No | 704 | 0.17 | 3365 | 0.83 | 1.586 | 1.354-1.858 | <.0001 | 1.464 | 1.224-1.751 | <.0001 | 311 | 0.18 | 1428 | 0.82 | 1.419 | 1.131-1.779 | 0.0025 |
| Yes | 108 | 0.17 | 522 | 0.83 | 1.338 | 0.883-2.028 | 0.1754 | 1.429 | 0.905-2.256 | 0.1258 | 55 | 0.21 | 212 | 0.79 | 1.127 | 0.664-1.913 | 0.6574 |
| Moderate or severe liver disease | Interaction: 0.301 | | | | | | | | | | Interaction: 0.745 | | | | | | |
| No | 801 | 0.17 | 3857 | 0.83 | 1.564 | 1.348-1.815 | <.0001 | 1.447 | 1.225-1.710 | <.0001 | 361 | 0.18 | 1622 | 0.82 | 1.382 | 1.121-1.704 | 0.0025 |
| Yes | 11 | 0.27 | 30 | 0.73 | 0.736 | 0.155-3.505 | 0.6983 | NA | NA | NA | 5 | 0.22 | 18 | 0.78 | 0.859 | 0.138-5.350 | 0.8705 |
| Metastatic solid tumor | Interaction: 0.716 | | | | | | | | | | Interaction: 0.329 | | | | | | |
| No | 801 | 0.17 | 3838 | 0.83 | 1.546 | 1.332-1.794 | <.0001 | 1.413 | 1.196-1.670 | <.0001 | 359 | 0.18 | 1615 | 0.82 | 1.356 | 1.099-1.673 | 0.0045 |
| Yes | 11 | 0.18 | 49 | 0.82 | 1.877 | 0.569-6.195 | 0.3172 | NA | NA | NA | 7 | 0.22 | 25 | 0.78 | 3.211 | 0.708-14.561 | 0.1303 |
| HIV | Interaction: NA | | | | | | | | | | Interaction: NA | | | | | | |
| No | 812 | 0.17 | 3886 | 0.83 | 1.552 | 1.338-1.799 | <.0001 | 1.427 | 1.209-1.684 | <.0001 | 366 | 0.18 | 1640 | 0.82 | 1.374 | 1.116-1.692 | 0.0028 |
| Yes | 0 | 0.00 | 1 | 1.00 | NA | NA | NA | NA | NA | NA | NA | NA | NA | NA | NA | NA | NA |
| Index year | Interaction: 0.060 | | | | | | | | | | Interaction: 0.069 | | | | | | |
| 2005 | 105 | 0.29 | 257 | 0.71 | 1.816 | 1.058-3.120 | 0.0254 | 2.040 | 1.158-3.593 | 0.0136 | 42 | 0.26 | 119 | 0.74 | 1.602 | 0.858-2.990 | 0.1391 |
| 2006 | 56 | 0.18 | 250 | 0.82 | 1.479 | 0.844-2.592 | 0.1648 | 1.504 | 0.798-2.834 | 0.2065 | 45 | 0.20 | 185 | 0.80 | 1.507 | 0.823-2.759 | 0.1835 |
| 2007 | 64 | 0.18 | 299 | 0.82 | 1.089 | 0.620-1.911 | 0.7717 | 1.091 | 0.593-2.008 | 0.7801 | 39 | 0.18 | 173 | 0.82 | 0.855 | 0.448-1.632 | 0.6358 |
| 2008 | 79 | 0.17 | 374 | 0.83 | 0.834 | 0.449-1.546 | 0.5766 | 0.726 | 0.371-1.422 | 0.3508 | 26 | 0.16 | 132 | 0.84 | 0.932 | 0.424-2.051 | 0.8615 |
| 2009 | 75 | 0.15 | 415 | 0.85 | 1.599 | 0.943-2.712 | 0.0813 | 1.594 | 0.892-2.849 | 0.1153 | 38 | 0.17 | 182 | 0.83 | 1.228 | 0.644-2.343 | 0.5332 |
| 2010 | 97 | 0.17 | 488 | 0.83 | 0.918 | 0.507-1.662 | 0.7770 | 0.919 | 0.493-1.712 | 0.7895 | 31 | 0.15 | 171 | 0.85 | 0.967 | 0.466-2.008 | 0.9287 |
| 2011 | 93 | 0.16 | 473 | 0.84 | 1.883 | 1.203-2.947 | 0.0063 | 1.617 | 0.994-2.632 | 0.0530 | 39 | 0.18 | 175 | 0.82 | 2.329 | 1.195-4.538 | 0.0130 |
| 2012 | 92 | 0.17 | 453 | 0.83 | 2.210 | 1.434-3.404 | 0.0004 | 2.525 | 1.586-4.020 | <.0001 | 41 | 0.20 | 167 | 0.80 | 2.931 | 1.490-5.767 | 0.0018 |
| 2013 | 66 | 0.15 | 381 | 0.85 | 1.459 | 0.820-2.595 | 0.2007 | 1.545 | 0.823-2.903 | 0.1760 | 29 | 0.18 | 130 | 0.82 | 1.024 | 0.493-2.125 | 0.9503 |
| 2014 | 71 | 0.15 | 394 | 0.85 | 0.878 | 0.482-1.599 | 0.6708 | 0.929 | 0.480-1.797 | 0.8266 | 29 | 0.15 | 162 | 0.85 | 0.817 | 0.393-1.700 | 0.5896 |
| 2015 | 14 | 0.12 | 103 | 0.88 | 2.014 | 0.679-5.974 | 0.2093 | 2.681 | 0.514-13.990 | 0.2421 | 7 | 0.14 | 44 | 0.86 | 2.562 | 0.497-13.214 | 0.2609 |

Abbreviations: HR, hazard ratio; CI, chronic obstructive pulmonary disease; COPD, modified Charlson comorbidity index; mCCI, human immunodeficiency virus; HIV, not applicable; NA.

**Table S10**. Hazard ratios of pneumonia-related hospitalization

| **Pneumonia-related hospitalization** | Unmatched population | | | | | | | | | | Propensity score-matched population | | | | | | |
| --- | --- | --- | --- | --- | --- | --- | --- | --- | --- | --- | --- | --- | --- | --- | --- | --- | --- |
|  | Event | | No event | | Crude HR | | | Adjusted HR | | | Event | | No event | | Crude HR | | |
|  | n | % | n | % | HR | 95% CI | p-value | HR | 95% CI | p-value | n | % | n | % | HR | 95% CI | p-value |
| Overall | 502 | 0.11 | 4197 | 0.89 | 1.883 | 1.567-2.262 | <.0001 | 1.618 | 1.317-1.989 | <.0001 | 233 | 0.12 | 1773 | 0.88 | 1.610 | 1.234-2.101 | 0.0005 |
| Age | Interaction: 0.256 | | | | | | | | | | Interaction: 0.890 | | | | | | |
| 55 to < 75 | 301 | 0.09 | 3074 | 0.91 | 2.024 | 1.597-2.565 | <.0001 | 1.638 | 1.250-2.145 | 0.0003 | 141 | 0.10 | 1283 | 0.90 | 1.540 | 1.097-2.163 | 0.0126 |
| 75+ | 201 | 0.15 | 1123 | 0.85 | 1.587 | 1.193-2.111 | 0.0015 | 1.592 | 1.147-2.210 | 0.0054 | 92 | 0.16 | 490 | 0.84 | 1.607 | 1.064-2.427 | 0.0240 |
| Sex | Interaction: 0.676 | | | | | | | | | | Interaction: 0.895 | | | | | | |
| Male | 414 | 0.11 | 3475 | 0.89 | 1.922 | 1.564-2.361 | <.0001 | 1.683 | 1.340-2.112 | <.0001 | 190 | 0.12 | 1341 | 0.88 | 1.599 | 1.192-2.146 | 0.0018 |
| Female | 88 | 0.11 | 722 | 0.89 | 1.789 | 1.167-2.743 | 0.0076 | 1.431 | 0.873-2.346 | 0.1554 | 43 | 0.09 | 432 | 0.91 | 1.681 | 0.897-3.150 | 0.1050 |
| Income level | Interaction: 0.918 | | | | | | | | | | Interaction: 0.884 | | | | | | |
| 1st quartile | 55 | 0.08 | 631 | 0.92 | 1.555 | 0.873-2.770 | 0.1343 | 1.338 | 0.700-2.556 | 0.3785 | 34 | 0.10 | 292 | 0.90 | 1.105 | 0.559-2.187 | 0.7733 |
| 2nd quartile | 71 | 0.11 | 580 | 0.89 | 1.857 | 1.100-3.135 | 0.0205 | 1.694 | 0.967-2.969 | 0.0653 | 34 | 0.13 | 220 | 0.87 | 1.747 | 0.896-3.407 | 0.1014 |
| 3rd quartile | 78 | 0.08 | 850 | 0.92 | 1.511 | 0.901-2.535 | 0.1180 | 1.503 | 0.865-2.612 | 0.1486 | 30 | 0.09 | 317 | 0.91 | 1.663 | 0.756-3.659 | 0.2062 |
| 4th quartile | 171 | 0.10 | 1482 | 0.90 | 1.910 | 1.368-2.669 | 0.0001 | 1.747 | 1.217-2.508 | 0.0025 | 74 | 0.11 | 603 | 0.89 | 1.922 | 1.185-3.117 | 0.0081 |
| Medical aid | 127 | 0.16 | 654 | 0.84 | 1.784 | 1.256-2.533 | 0.0012 | 1.424 | 0.926-2.190 | 0.1072 | 61 | 0.15 | 341 | 0.85 | 1.543 | 0.924-2.577 | 0.0975 |
| Hospital type | Interaction: 0.857 | | | | | | | | | | Interaction: 0.822 | | | | | | |
| General hospital | 439 | 0.11 | 3597 | 0.89 | 1.880 | 1.543-2.291 | <.0001 | 1.577 | 1.260-1.973 | <.0001 | 206 | 0.12 | 1501 | 0.88 | 1.556 | 1.173-2.065 | 0.0022 |
| Hospital | 32 | 0.13 | 219 | 0.87 | 1.585 | 0.767-3.276 | 0.2135 | 1.761 | 0.734-4.230 | 0.2053 | 15 | 0.11 | 119 | 0.89 | 3.897 | 1.236-12.288 | 0.0203 |
| Clinic | 31 | 0.08 | 363 | 0.92 | 3.060 | 1.474-6.355 | 0.0027 | 1.400 | 0.533-3.679 | 0.4944 | 12 | 0.08 | 146 | 0.92 | 1.358 | 0.421-4.386 | 0.6087 |
| Others | 0 | 0.00 | 18 | 1.00 | NA | NA | NA | NA | NA | NA | 0 | 0.00 | 7 | 1.00 | NA | NA | NA |
| History of COPD exacerbation | Interaction: 0.505 | | | | | | | | | | Interaction: 0.337 | | | | | | |
| None | 397 | 0.10 | 3559 | 0.90 | 2.001 | 1.631-2.456 | <.0001 | 1.647 | 1.301-2.084 | <.0001 | 182 | 0.11 | 1469 | 0.89 | 1.607 | 1.188-2.172 | 0.0021 |
| 1 moderate | 40 | 0.13 | 280 | 0.88 | 2.058 | 1.045-4.051 | 0.0367 | 2.271 | 1.010-5.105 | 0.0472 | 17 | 0.12 | 128 | 0.88 | 3.947 | 1.281-12.163 | 0.0168 |
| ≥ 2 moderate OR ≥ 1 severe | 65 | 0.15 | 358 | 0.85 | 1.226 | 0.726-2.072 | 0.4462 | 1.563 | 0.865-2.825 | 0.1391 | 34 | 0.16 | 176 | 0.84 | 1.127 | 0.576-2.201 | 0.7274 |
| History of pneumonia | Interaction: 0.236 | | | | | | | | | | Interaction: 0.588 | | | | | | |
| No | 430 | 0.10 | 3808 | 0.90 | 2.024 | 1.664-2.463 | <.0001 | 1.654 | 1.326-2.064 | <.0001 | 204 | 0.11 | 1627 | 0.89 | 1.663 | 1.250-2.211 | 0.0005 |
| Yes | 72 | 0.16 | 389 | 0.84 | 1.337 | 0.769-2.324 | 0.3038 | 1.291 | 0.701-2.380 | 0.4128 | 29 | 0.17 | 146 | 0.83 | 1.301 | 0.616-2.746 | 0.4898 |
| mCCI | Interaction: 0.712 | | | | | | | | | | Interaction: 0.897 | | | | | | |
| 0,1 | 282 | 0.10 | 2412 | 0.90 | 2.164 | 1.706-2.747 | <.0001 | 1.651 | 1.247-2.185 | 0.0005 | 132 | 0.11 | 1018 | 0.89 | 1.570 | 1.103-2.235 | 0.0122 |
| 2 | 49 | 0.09 | 518 | 0.91 | 1.451 | 0.744-2.827 | 0.2746 | 1.926 | 0.934-3.971 | 0.0757 | 23 | 0.10 | 213 | 0.90 | 1.218 | 0.524-2.831 | 0.6475 |
| 3 | 83 | 0.11 | 697 | 0.89 | 1.679 | 1.041-2.708 | 0.0335 | 1.656 | 0.972-2.821 | 0.0636 | 38 | 0.12 | 274 | 0.88 | 1.714 | 0.904-3.249 | 0.0988 |
| 4+ | 88 | 0.13 | 570 | 0.87 | 1.440 | 0.914-2.269 | 0.1159 | 1.463 | 0.893-2.397 | 0.1307 | 40 | 0.13 | 268 | 0.87 | 1.772 | 0.925-3.395 | 0.0846 |
| mCCI category |  | | | | | | | | | |  | | | | | | |
| Congestive heart failure | Interaction: 0.332 | | | | | | | | | | Interaction: 0.093 | | | | | | |
| No | 445 | 0.10 | 3795 | 0.90 | 1.835 | 1.509-2.231 | <.0001 | 1.539 | 1.234-1.920 | 0.0001 | 209 | 0.12 | 1590 | 0.88 | 1.487 | 1.125-1.966 | 0.0053 |
| Yes | 57 | 0.12 | 402 | 0.88 | 2.348 | 1.379-3.996 | 0.0017 | 2.657 | 1.401-5.038 | 0.0028 | 24 | 0.12 | 183 | 0.88 | 3.819 | 1.368-10.662 | 0.0105 |
| Dementia | Interaction: 0.840 | | | | | | | | | | Interaction: 0.912 | | | | | | |
| No | 483 | 0.11 | 4051 | 0.89 | 1.906 | 1.581-2.298 | <.0001 | 1.616 | 1.310-1.994 | <.0001 | 225 | 0.12 | 1711 | 0.88 | 1.607 | 1.225-2.107 | 0.0006 |
| Yes | 19 | 0.12 | 146 | 0.88 | 1.336 | 0.491-3.636 | 0.5701 | 2.419 | 0.644-9.085 | 0.1908 | 8 | 0.11 | 62 | 0.89 | 1.667 | 0.397-7.003 | 0.4849 |
| Chronic pulmonary disease | Interaction 0.668 | | | | | | | | | | Interaction: 0.707 | | | | | | |
| No | 214 | 0.10 | 1872 | 0.90 | 2.150 | 1.641-2.817 | <.0001 | 1.575 | 1.135-2.185 | 0.0065 | 103 | 0.12 | 786 | 0.88 | 1.461 | 0.984-2.170 | 0.0600 |
| Yes | 288 | 0.11 | 2325 | 0.89 | 1.739 | 1.346-2.246 | <.0001 | 1.702 | 1.296-2.234 | 0.0001 | 130 | 0.12 | 987 | 0.88 | 1.841 | 1.273-2.662 | 0.0012 |
| Rheumatologic disease | Interaction: 0.101 | | | | | | | | | | Interaction: 0.719 | | | | | | |
| No | 479 | 0.11 | 4049 | 0.89 | 1.947 | 1.614-2.348 | <.0001 | 1.677 | 1.358-2.073 | <.0001 | 223 | 0.12 | 1680 | 0.88 | 1.624 | 1.238-2.130 | 0.0005 |
| Yes | 23 | 0.13 | 148 | 0.87 | 0.792 | 0.320-1.960 | 0.6140 | 0.545 | 0.164-1.816 | 0.3231 | 10 | 0.10 | 93 | 0.90 | 1.247 | 0.333-4.670 | 0.7435 |
| Mild liver disease | Interaction: 0.165 | | | | | | | | | | Interaction: 0.274 | | | | | | |
| No | 395 | 0.11 | 3325 | 0.89 | 2.061 | 1.681-2.527 | <.0001 | 1.721 | 1.362-2.175 | <.0001 | 179 | 0.11 | 1405 | 0.89 | 1.744 | 1.280-2.376 | 0.0004 |
| Yes | 107 | 0.11 | 872 | 0.89 | 1.327 | 0.859-2.050 | 0.2023 | 1.326 | 0.837-2.100 | 0.2298 | 54 | 0.13 | 368 | 0.87 | 1.265 | 0.741-2.158 | 0.3894 |
| Diabetes with chronic complications | Interaction: 0.551 | | | | | | | | | | Interaction: 0.419 | | | | | | |
| No | 441 | 0.10 | 3787 | 0.90 | 1.926 | 1.585-2.341 | <.0001 | 1.609 | 1.289-2.009 | <.0001 | 207 | 0.11 | 1596 | 0.89 | 1.535 | 1.158-2.035 | 0.0029 |
| Yes | 61 | 0.13 | 410 | 0.87 | 1.647 | 0.954-2.842 | 0.0733 | 1.700 | 0.900-3.209 | 0.1018 | 26 | 0.13 | 177 | 0.87 | 2.516 | 1.068-5.928 | 0.0348 |
| Hemiplegia or paraplegia | Interaction: 0.857 | | | | | | | | | | Interaction: 0.793 | | | | | | |
| No | 496 | 0.11 | 4151 | 0.89 | 1.880 | 1.563-2.261 | <.0001 | 1.618 | 1.314-1.992 | <.0001 | 230 | 0.12 | 1756 | 0.88 | 1.603 | 1.227-2.096 | 0.0006 |
| Yes | 6 | 0.12 | 46 | 0.88 | 2.045 | 0.379-11.020 | 0.44051 | NA | NA | NA | 3 | 0.15 | 17 | 0.85 | 1.808 | 0.180-18.168 | 0.6150 |
| Renal disease | Interaction: 0.378 | | | | | | | | | | Interaction: 0.594 | | | | | | |
| No | 484 | 0.11 | 4085 | 0.89 | 1.921 | 1.594-2.315 | <.0001 | 1.641 | 1.330-2.024 | <.0001 | 223 | 0.12 | 1707 | 0.88 | 1.632 | 1.243-2.143 | 0.0004 |
| Yes | 18 | 0.14 | 112 | 0.86 | 1.038 | 0.373-2.885 | 0.9429 | 0.793 | 0.122-5.134 | 0.8077 | 10 | 0.13 | 66 | 0.87 | 1.220 | 0.343-4.348 | 0.7586 |
| Any malignancy, including lymphoma and leukemia | Interaction: 0.267 | | | | | | | | | | Interaction: 0.564 | | | | | | |
| No | 433 | 0.11 | 3636 | 0.89 | 1.989 | 1.635-2.421 | <.0001 | 1.700 | 1.360-2.127 | <.0001 | 200 | 0.12 | 1539 | 0.88 | 1.675 | 1.256-2.234 | 0.0004 |
| Yes | 69 | 0.11 | 561 | 0.89 | 1.328 | 0.786-2.244 | 0.2894 | 1.402 | 0.791-2.483 | 0.2472 | 33 | 0.12 | 234 | 0.88 | 1.278 | 0.644-2.537 | 0.4823 |
| Moderate or severe liver disease | Interaction: 0.452 | | | | | | | | | | Interaction: 0.545 | | | | | | |
| No | 496 | 0.11 | 4162 | 0.89 | 1.899 | 1.579-2.283 | <.0001 | 1.638 | 1.331-2.015 | <.0001 | 230 | 0.12 | 1753 | 0.88 | 1.628 | 1.245-2.128 | 0.0004 |
| Yes | 6 | 0.15 | 35 | 0.85 | 0.936 | 0.091-9.605 | 0.9558 | NA | NA | NA | 3 | 0.13 | 20 | 0.87 | 0.735 | 0.041-13.291 | 0.8351 |
| Metastatic solid tumor | Interaction: 0.626 | | | | | | | | | | Interaction: 0.866 | | | | | | |
| No | 495 | 0.11 | 4144 | 0.89 | 1.900 | 1.579-2.285 | <.0001 | 1.628 | 1.322-2.004 | <.0001 | 229 | 0.12 | 1745 | 0.88 | 1.612 | 1.231-2.110 | 0.0005 |
| Yes | 7 | 0.12 | 53 | 0.88 | 1.300 | 0.260-6.497 | 0.7491 | NA | NA | NA | 4 | 0.13 | 28 | 0.88 | 2.058 | 0.334-12.664 | 0.4362 |
| HIV | Interaction: NA | | | | | | | | | | Interaction: NA | | | | | | |
| No | 502 | 0.11 | 4196 | 0.89 | 1.885 | 1.569-2.265 | <.0001 | 1.618 | 1.317-1.989 | <.0001 | 233 | 0.12 | 1773 | 0.88 | 1.610 | 1.234-2.102 | 0.0005 |
| Yes | 0 | 0.00 | 1 | 1.00 | NA | NA | NA | NA | NA | NA | NA | NA | NA | NA | NA | NA | NA |
| Index year | Interaction: 0.780 | | | | | | | | | | Interaction: 0.483 | | | | | | |
| 2005 | 78 | 0.22 | 284 | 0.78 | 1.720 | 0.921-3.211 | 0.0886 | 1.524 | 0.809-2.871 | 0.1923 | 30 | 0.19 | 131 | 0.81 | 1.554 | 0.751-3.216 | 0.2347 |
| 2006 | 35 | 0.11 | 271 | 0.89 | 2.159 | 1.061-4.391 | 0.0336 | 3.062 | 1.313-7.140 | 0.0096 | 31 | 0.13 | 199 | 0.87 | 1.875 | 0.903-3.891 | 0.0917 |
| 2007 | 42 | 0.12 | 321 | 0.88 | 1.229 | 0.619-2.440 | 0.5553 | 1.094 | 0.520-2.302 | 0.8118 | 25 | 0.12 | 187 | 0.88 | 1.017 | 0.465-2.224 | 0.9656 |
| 2008 | 42 | 0.09 | 411 | 0.91 | 1.440 | 0.709-2.928 | 0.3134 | 1.488 | 0.673-3.291 | 0.3259 | 18 | 0.11 | 140 | 0.89 | 1.226 | 0.477-3.155 | 0.6720 |
| 2009 | 43 | 0.09 | 447 | 0.91 | 1.617 | 0.816-3.202 | 0.1681 | 2.002 | 0.931-4.307 | 0.0757 | 21 | 0.10 | 199 | 0.90 | 1.263 | 0.544-2.932 | 0.5862 |
| 2010 | 66 | 0.11 | 519 | 0.89 | 1.082 | 0.547-2.138 | 0.8216 | 0.861 | 0.409-1.812 | 0.6936 | 21 | 0.10 | 181 | 0.90 | 1.226 | 0.511-2.946 | 0.6480 |
| 2011 | 53 | 0.09 | 513 | 0.91 | 2.457 | 1.399-4.316 | 0.0018 | 2.004 | 1.079-3.723 | 0.0277 | 21 | 0.10 | 193 | 0.90 | 7.105 | 2.111-23.915 | 0.0015 |
| 2012 | 56 | 0.10 | 489 | 0.90 | 1.853 | 1.042-3.295 | 0.0359 | 1.866 | 1.015-3.432 | 0.0448 | 26 | 0.13 | 182 | 0.88 | 1.847 | 0.831-4.103 | 0.1320 |
| 2013 | 38 | 0.09 | 409 | 0.91 | 2.088 | 1.029-4.237 | 0.0415 | 2.093 | 0.962-4.554 | 0.0627 | 18 | 0.11 | 141 | 0.89 | 1.554 | 0.590-4.094 | 0.3727 |
| 2014 | 39 | 0.08 | 426 | 0.92 | 1.182 | 0.564-2.477 | 0.6579 | 1.624 | 0.717-3.680 | 0.2452 | 18 | 0.09 | 173 | 0.91 | 1.003 | 0.397-2.537 | 0.9945 |
| 2015 | 10 | 0.09 | 107 | 0.91 | 1.487 | 0.394-5.608 | 0.5579 | 3.496 | 0.337-36.250 | 0.2943 | 4 | 0.08 | 47 | 0.92 | 2.883 | 0.300-27.742 | 0.3594 |

Abbreviations: HR, hazard ratio; CI, chronic obstructive pulmonary disease; COPD, modified Charlson comorbidity index; mCCI, human immunodeficiency virus; HIV, not applicable; NA.

**Table S11**. Hazard ratios of outpatient pneumonia events

| **Outpatient pneumonia events** | Unmatched population | | | | | | | | | | Propensity score-matched population | | | | | | |
| --- | --- | --- | --- | --- | --- | --- | --- | --- | --- | --- | --- | --- | --- | --- | --- | --- | --- |
|  | Event | | No event | | Crude HR | | | Adjusted HR | | | Event | | No event | | Crude HR | | |
|  | n | % | n | % | HR | 95% CI | p-value | HR | 95% CI | p-value | n | % | n | % | HR | 95% CI | p-value |
| Overall | 442 | 0.09 | 4257 | 0.91 | 1.266 | 1.029-1.559 | 0.0258 | 1.274 | 1.012-1.604 | 0.0395 | 188 | 0.09 | 1818 | 0.91 | 1.231 | 0.926-1.637 | 0.1522 |
| Age | Interaction: 0.703 | | | | | | | | | | Interaction: 0.476 | | | | | | |
| 55 to < 75 | 313 | 0.09 | 3062 | 0.91 | 1.299 | 1.014-1.664 | 0.0383 | 1.317 | 1.002-1.731 | 0.0487 | 133 | 0.09 | 1291 | 0.91 | 1.307 | 0.927-1.841 | 0.1264 |
| 75+ | 129 | 0.10 | 1195 | 0.90 | 1.168 | 0.800-1.708 | 0.4212 | 1.100 | 0.711-1.702 | 0.6689 | 55 | 0.09 | 527 | 0.91 | 1.058 | 0.626-1.789 | 0.8326 |
| Sex | Interaction: 0.997 | | | | | | | | | | Interaction: 0.518 | | | | | | |
| Male | 352 | 0.09 | 3537 | 0.91 | 1.233 | 0.968-1.571 | 0.0897 | 1.244 | 0.954-1.621 | 0.1067 | 141 | 0.09 | 1390 | 0.91 | 1.163 | 0.834-1.621 | 0.3725 |
| Female | 90 | 0.11 | 720 | 0.89 | 1.245 | 0.820-1.891 | 0.3033 | 1.334 | 0.822-2.164 | 0.2441 | 47 | 0.10 | 428 | 0.90 | 1.465 | 0.825-2.622 | 0.1919 |
| Income level | Interaction: 0.987 | | | | | | | | | | Interaction: 0.990 | | | | | | |
| 1st quartile | 58 | 0.08 | 628 | 0.92 | 1.130 | 0.627-2.034 | 0.6848 | 1.113 | 0.571-2.167 | 0.7534 | 28 | 0.09 | 298 | 0.91 | 1.152 | 0.550-2.413 | 0.7066 |
| 2nd quartile | 53 | 0.08 | 598 | 0.92 | 1.310 | 0.701-2.450 | 0.3971 | 1.252 | 0.619-2.531 | 0.5324 | 20 | 0.08 | 234 | 0.92 | 1.425 | 0.596-3.404 | 0.4255 |
| 3rd quartile | 101 | 0.11 | 827 | 0.89 | 1.306 | 0.817-2.088 | 0.2640 | 1.380 | 0.831-2.293 | 0.2137 | 45 | 0.13 | 302 | 0.87 | 1.143 | 0.625-2.088 | 0.6649 |
| 4th quartile | 151 | 0.09 | 1502 | 0.91 | 1.191 | 0.808-1.754 | 0.3768 | 1.133 | 0.754-1.702 | 0.5487 | 56 | 0.08 | 621 | 0.92 | 1.356 | 0.806-2.283 | 0.2512 |
| Medical aid | 79 | 0.10 | 702 | 0.90 | 1.432 | 0.923-2.223 | 0.1095 | 1.184 | 0.680-2.061 | 0.5506 | 39 | 0.10 | 363 | 0.90 | 1.276 | 0.677-2.405 | 0.4506 |
| Hospital type | Interaction: 0.387 | | | | | | | | | | Interaction: 0.985 | | | | | | |
| General hospital | 389 | 0.10 | 3647 | 0.90 | 1.338 | 1.073-1.668 | 0.0097 | 1.340 | 1.050-1.711 | 0.0187 | 168 | 0.10 | 1539 | 0.90 | 1.252 | 0.923-1.698 | 0.1483 |
| Hospital | 15 | 0.06 | 236 | 0.94 | 0.356 | 0.080-1.576 | 0.1735 | 0.604 | 0.096-3.820 | 0.5921 | 4 | 0.03 | 130 | 0.97 | 0.919 | 0.138-6.117 | 0.9304 |
| Clinic | 36 | 0.09 | 358 | 0.91 | 1.234 | 0.584-2.605 | 0.5815 | 0.907 | 0.355-2.317 | 0.8379 | 15 | 0.09 | 143 | 0.91 | 1.242 | 0.445-3.470 | 0.6793 |
| Others | 2 | 0.11 | 16 | 0.89 | NA | NA | NA | NA | NA | NA | 1 | 0.14 | 6 | 0.86 | NA | NA | NA |
| History of COPD exacerbation | Interaction: 0.787 | | | | | | | | | | Interaction: 0.263 | | | | | | |
| None | 346 | 0.09 | 3610 | 0.91 | 1.289 | 1.020-1.628 | 0.0334 | 1.193 | 0.913-1.560 | 0.1966 | 146 | 0.09 | 1505 | 0.91 | 1.137 | 0.822-1.573 | 0.4368 |
| 1 moderate | 39 | 0.12 | 281 | 0.88 | 1.689 | 0.841-3.391 | 0.1405 | 2.165 | 0.942-4.972 | 0.0688 | 17 | 0.12 | 128 | 0.88 | 3.939 | 1.289-12.039 | 0.0162 |
| ≥ 2 moderate OR ≥ 1 severe | 57 | 0.13 | 366 | 0.87 | 0.950 | 0.521-1.732 | 0.8677 | 1.038 | 0.531-2.029 | 0.9136 | 25 | 0.12 | 185 | 0.88 | 1.092 | 0.491-2.428 | 0.8290 |
| History of pneumonia | Interaction: 0.043 | | | | | | | | | | Interaction: 0.698 | | | | | | |
| No | 330 | 0.08 | 3908 | 0.92 | 1.551 | 1.232-1.954 | 0.0002 | 1.438 | 1.109-1.864 | 0.0061 | 154 | 0.08 | 1677 | 0.92 | 1.296 | 0.945-1.776 | 0.1072 |
| Yes | 112 | 0.24 | 349 | 0.76 | 0.732 | 0.439-1.219 | 0.2302 | 0.717 | 0.413-1.246 | 0.2381 | 34 | 0.19 | 141 | 0.81 | 0.934 | 0.465-1.875 | 0.8474 |
| mCCI | Interaction: 0.856 | | | | | | | | | | Interaction: 0.875 | | | | | | |
| 0,1 | 260 | 0.10 | 2434 | 0.90 | 1.188 | 0.909-1.552 | 0.2078 | 1.203 | 0.884-1.638 | 0.2405 | 108 | 0.09 | 1042 | 0.91 | 1.204 | 0.826-1.755 | 0.3351 |
| 2 | 39 | 0.07 | 528 | 0.93 | 1.519 | 0.756-3.054 | 0.2403 | 1.537 | 0.725-3.259 | 0.2619 | 20 | 0.08 | 216 | 0.92 | 1.172 | 0.481-2.852 | 0.7269 |
| 3 | 80 | 0.10 | 700 | 0.90 | 1.377 | 0.828-2.292 | 0.2181 | 1.421 | 0.820-2.462 | 0.2102 | 33 | 0.11 | 279 | 0.89 | 1.079 | 0.543-2.146 | 0.8274 |
| 4+ | 63 | 0.10 | 595 | 0.90 | 1.261 | 0.730-2.177 | 0.4059 | 1.305 | 0.726-2.346 | 0.3742 | 27 | 0.09 | 281 | 0.91 | 1.567 | 0.703-3.410 | 0.2575 |
| mCCI category |  | | | | | | | | | |  | | | | | | |
| Congestive heart failure | Interaction: 0.378 | | | | | | | | | | Interaction: 0.776 | | | | | | |
| No | 408 | 0.10 | 3832 | 0.90 | 1.226 | 0.987-1.523 | 0.0651 | 1.245 | 0.978-1.584 | 0.0752 | 174 | 0.10 | 1625 | 0.90 | 1.217 | 0.904-1.639 | 0.1962 |
| Yes | 34 | 0.07 | 425 | 0.93 | 1.772 | 0.861-3.647 | 0.1201 | 1.183 | 0.475-2.945 | 0.7188 | 14 | 0.07 | 193 | 0.93 | 1.431 | 0.478-4.288 | 0.5219 |
| Dementia | Interaction: 0.168 | | | | | | | | | | Interaction: 0.261 | | | | | | |
| No | 425 | 0.09 | 4109 | 0.91 | 1.302 | 1.055-1.607 | 0.0140 | 1.313 | 1.039-1.669 | 0.0224 | 182 | 0.09 | 1754 | 0.91 | 1.270 | 0.948-1.702 | 0.1096 |
| Yes | 17 | 0.10 | 148 | 0.90 | 0.472 | 0.107-2.088 | 0.3222 | 0.600 | 0.082-4.367 | 0.6141 | 6 | 0.09 | 64 | 0.91 | 0.487 | 0.094-2.524 | 0.3912 |
| Chronic pulmonary disease | Interaction 0.996 | | | | | | | | | | Interaction: 0.804 | | | | | | |
| No | 163 | 0.08 | 1923 | 0.92 | 1.355 | 0.980-1.872 | 0.0657 | 1.197 | 0.804-1.782 | 0.3756 | 70 | 0.08 | 819 | 0.92 | 1.157 | 0.726-1.844 | 0.5385 |
| Yes | 279 | 0.11 | 2334 | 0.89 | 1.283 | 0.976-1.688 | 0.0746 | 1.292 | 0.968-1.724 | 0.0816 | 118 | 0.11 | 999 | 0.89 | 1.321 | 0.913-1.909 | 0.1394 |
| Rheumatologic disease | Interaction: 0.108 | | | | | | | | | | Interaction: 0.040 | | | | | | |
| No | 423 | 0.09 | 4105 | 0.91 | 1.225 | 0.989-1.517 | 0.0636 | 1.199 | 0.940-1.516 | 0.1453 | 179 | 0.09 | 1724 | 0.91 | 1.143 | 0.851-1.534 | 0.3753 |
| Yes | 19 | 0.11 | 152 | 0.89 | 2.263 | 0.931-5.497 | 0.0714 | 1.954 | 0.459-8.312 | 0.3643 | 9 | 0.09 | 94 | 0.91 | 8.748 | 1.072-71.374 | 0.0429 |
| Mild liver disease | Interaction: 0.370 | | | | | | | | | | Interaction: 0.757 | | | | | | |
| No | 348 | 0.09 | 3372 | 0.91 | 1.324 | 1.052-1.666 | 0.0167 | 1.333 | 1.028-1.729 | 0.0304 | 152 | 0.10 | 1432 | 0.90 | 1.247 | 0.907-1.715 | 0.1736 |
| Yes | 94 | 0.10 | 885 | 0.90 | 1.062 | 0.651-1.730 | 0.8106 | 1.118 | 0.666-1.878 | 0.6727 | 36 | 0.09 | 386 | 0.91 | 1.185 | 0.607-2.313 | 0.6198 |
| Diabetes with chronic complications | Interaction: 0.614 | | | | | | | | | | Interaction: 0.855 | | | | | | |
| No | 401 | 0.09 | 3827 | 0.91 | 1.255 | 1.009-1.561 | 0.0413 | 1.232 | 0.966-1.572 | 0.0934 | 169 | 0.09 | 1634 | 0.91 | 1.227 | 0.906-1.662 | 0.1862 |
| Yes | 41 | 0.10 | 430 | 0.91 | 1.359 | 0.689-2.683 | 0.3759 | 1.742 | 0.823-3.684 | 0.1466 | 19 | 0.09 | 184 | 0.91 | 1.242 | 0.501-3.080 | 0.6403 |
| Hemiplegia or paraplegia | Interaction: 0.826 | | | | | | | | | | Interaction: 0.896 | | | | | | |
| No | 438 | 0.09 | 4209 | 0.91 | 1.262 | 1.025-1.555 | 0.0285 | 1.265 | 1.003-1.595 | 0.0467 | 186 | 0.09 | 1800 | 0.91 | 1.232 | 0.924-1.645 | 0.1557 |
| Yes | 4 | 0.08 | 48 | 0.92 | 2.287 | 0.176-29.755 | 0.5275 | 1.934 | NA | 1.0000 | 2 | 0.10 | 18 | 0.90 | 0.904 | 0.065-13.279 | 0.9416 |
| Renal disease | Interaction: 0.745 | | | | | | | | | | Interaction: 0.530 | | | | | | |
| No | 437 | 0.10 | 4132 | 0.90 | 1.280 | 1.039-1.577 | 0.0203 | 1.282 | 1.017-1.616 | 0.0355 | 185 | 0.10 | 1745 | 0.90 | 1.248 | 0.934-1.667 | 0.1344 |
| Yes | 5 | 0.04 | 125 | 0.96 | 0.578 | 0.066-5.045 | 0.6202 | NA | NA | NA | 3 | 0.04 | 73 | 0.96 | 0.513 | 0.049-5.428 | 0.5795 |
| Any malignancy, including lymphoma and leukemia | Interaction: 0.384 | | | | | | | | | | Interaction: 0.693 | | | | | | |
| No | 389 | 0.10 | 3680 | 0.90 | 1.218 | 0.975-1.522 | 0.0827 | 1.234 | 0.962-1.583 | 0.0982 | 159 | 0.09 | 1580 | 0.91 | 1.206 | 0.882-1.649 | 0.2411 |
| Yes | 53 | 0.08 | 577 | 0.92 | 1.669 | 0.939-2.967 | 0.0809 | 1.905 | 1.006-3.605 | 0.0478 | 29 | 0.11 | 238 | 0.89 | 1.385 | 0.671-2.859 | 0.3780 |
| Moderate or severe liver disease | Interaction: 0.323 | | | | | | | | | | Interaction: 0.841 | | | | | | |
| No | 436 | 0.09 | 4222 | 0.91 | 1.277 | 1.037-1.574 | 0.0216 | 1.295 | 1.027-1.634 | 0.0291 | 186 | 0.09 | 1797 | 0.91 | 1.229 | 0.921-1.641 | 0.1618 |
| Yes | 6 | 0.15 | 35 | 0.85 | 0.624 | 0.071-5.476 | 0.6706 | NA | NA | NA | 2 | 0.09 | 21 | 0.91 | 1.144 | 0.081-16.252 | 0.9208 |
| Metastatic solid tumor | Interaction: 0.027 | | | | | | | | | | Interaction: 0.081 | | | | | | |
| No | 436 | 0.09 | 4203 | 0.91 | 1.232 | 0.998-1.520 | 0.0519 | 1.226 | 0.971-1.549 | 0.0868 | 183 | 0.09 | 1791 | 0.91 | 1.180 | 0.882-1.579 | 0.2662 |
| Yes | 6 | 0.10 | 54 | 0.90 | 6.629 | 1.262-34.828 | 0.0255 | NA | NA | NA | 5 | 0.16 | 27 | 0.84 | 8.702 | 1.249-60.610 | 0.0289 |
| HIV | Interaction: NA | | | | | | | | | | Interaction: NA | | | | | | |
| No | 442 | 0.09 | 4256 | 0.91 | 1.268 | 1.030-1.560 | 0.0251 | 1.274 | 1.012-1.604 | 0.0395 | 188 | 0.09 | 1818 | 0.91 | 1.231 | 0.924-1.641 | 0.1559 |
| Yes | 0 | 0.00 | 1 | 1.00 | NA | NA | NA | NA | NA | NA | NA | NA | NA | NA | NA | NA | NA |
| Index year | Interaction: 0.110 | | | | | | | | | | Interaction: 0.062 | | | | | | |
| 2005 | 47 | 0.13 | 315 | 0.87 | 2.239 | 0.998-5.023 | 0.0507 | 3.209 | 1.212-8.495 | 0.0189 | 19 | 0.12 | 142 | 0.88 | 2.105 | 0.868-5.104 | 0.0994 |
| 2006 | 28 | 0.09 | 278 | 0.91 | 1.461 | 0.676-3.156 | 0.3348 | 1.252 | 0.538-2.910 | 0.6109 | 20 | 0.09 | 210 | 0.91 | 1.967 | 0.763-5.066 | 0.1613 |
| 2007 | 32 | 0.09 | 331 | 0.91 | 0.900 | 0.376-2.156 | 0.8129 | 0.670 | 0.254-1.769 | 0.4185 | 19 | 0.09 | 193 | 0.91 | 0.768 | 0.289-2.042 | 0.5971 |
| 2008 | 54 | 0.12 | 399 | 0.88 | 0.768 | 0.351-1.680 | 0.5083 | 0.632 | 0.273-1.461 | 0.2828 | 14 | 0.09 | 144 | 0.91 | 1.307 | 0.476-3.593 | 0.6032 |
| 2009 | 47 | 0.10 | 443 | 0.90 | 1.202 | 0.595-2.430 | 0.6082 | 1.076 | 0.493-2.349 | 0.8534 | 24 | 0.11 | 196 | 0.89 | 0.877 | 0.386-1.991 | 0.7534 |
| 2010 | 46 | 0.08 | 539 | 0.92 | 0.584 | 0.209-1.631 | 0.3047 | 0.671 | 0.232-1.941 | 0.4613 | 13 | 0.06 | 189 | 0.94 | 0.565 | 0.182-1.748 | 0.3215 |
| 2011 | 55 | 0.10 | 511 | 0.90 | 1.677 | 0.913-3.082 | 0.0957 | 1.484 | 0.774-2.847 | 0.2348 | 25 | 0.12 | 189 | 0.88 | 1.429 | 0.646-3.159 | 0.3781 |
| 2012 | 46 | 0.08 | 499 | 0.92 | 2.173 | 1.171-4.032 | 0.0139 | 3.356 | 1.692-6.655 | 0.0005 | 18 | 0.09 | 190 | 0.91 | 5.629 | 1.666-19.019 | 0.0054 |
| 2013 | 40 | 0.09 | 407 | 0.91 | 1.036 | 0.457-2.346 | 0.9333 | 1.218 | 0.496-2.988 | 0.6666 | 18 | 0.11 | 141 | 0.89 | 0.622 | 0.241-1.606 | 0.3264 |
| 2014 | 43 | 0.09 | 422 | 0.91 | 0.631 | 0.267-1.490 | 0.2934 | 0.567 | 0.208-1.546 | 0.2673 | 15 | 0.08 | 176 | 0.92 | 0.677 | 0.245-1.875 | 0.4531 |
| 2015 | 4 | 0.03 | 113 | 0.97 | 3.590 | 0.506-25.465 | 0.2009 | NA | NA | NA | 3 | 0.06 | 48 | 0.94 | 2.046 | 0.208-20.096 | 0.5393 |

Abbreviations: HR, hazard ratio; CI, chronic obstructive pulmonary disease; COPD, modified Charlson comorbidity index; mCCI, human immunodeficiency virus; HIV, not applicable; NA.

**Table S12**. Hazard ratios of pneumonia-related death

| **Pneumonia-related death** | Unmatched population | | | | | | | | | | Propensity score-matched population | | | | | | |
| --- | --- | --- | --- | --- | --- | --- | --- | --- | --- | --- | --- | --- | --- | --- | --- | --- | --- |
|  | Event | | No event | | Crude HR | | | Adjusted HR | | | Event | | No event | | Crude HR | | |
|  | n | % | n | % | HR | 95% CI | p-value | HR | 95% CI | p-value | n | % | n | % | HR | 95% CI | p-value |
| Overall | 23 | 0.00 | 4676 | 1.00 | 1.751 | 0.734-4.181 | 0.2068 | 1.902 | 0.736-4.912 | 0.1843 | 11 | 0.01 | 1995 | 0.99 | 2.432. | 0.642-9.209 | 0.1907 |
| Age | Interaction: 0.299 | | | | | | | | | | Interaction: 0.700 | | | | | | |
| 55 to < 75 | 12 | 0.00 | 3363 | 1.00 | 2.437 | 0.764-7.773 | 0.1322 | 3.053 | 0.791-11.780 | 0.1052 | 7 | 0.00 | 1417 | 1.00 | 2.041 | 0.380-10.968 | 0.4054 |
| 75+ | 11 | 0.01 | 1313 | 0.99 | 1.096 | 0.289-4.150 | 0.8930 | 1.424 | 0.298-6.803 | 0.6581 | 4 | 0.01 | 578 | 0.99 | 3.113 | 0.320-30.270 | 0.3278 |
| Sex | Interaction: 0.965 | | | | | | | | | | Interaction: 0.993 | | | | | | |
| Male | 19 | 0.00 | 3870 | 1.00 | 1.797 | 0.677-4.771 | 0.2393 | 2.094 | 0.728-6.021 | 0.1701 | 10 | 0.01 | 1521 | 0.99 | 2.166 | 0.540-8.690 | 0.2756 |
| Female | 4 | 0.00 | 806 | 1.00 | 1.714 | 0.209-14.046 | 0.6155 | NA | NA | NA | 1 | 0.00 | 474 | 1.00 | NA | NA | NA |
| Income level | Interaction: 0.732 | | | | | | | | | | Interaction: 0.946 | | | | | | |
| 1st quartile | 5 | 0.01 | 681 | 0.99 | 0.806 | 0.099-6.566 | 0.8400 | NA | NA | NA | 3 | 0.01 | 323 | 0.99 | 0.631 | 0.044-9.044 | 0.7343 |
| 2nd quartile | 4 | 0.01 | 647 | 0.99 | 3.865 | 0.503-29.707 | 0.1938 | NA | NA | NA | 3 | 0.01 | 251 | 0.99 | 2.119 | 0.181-24.840 | 0.5500 |
| 3rd quartile | 4 | 0.00 | 924 | 1.00 | 1.358 | 0.147-12.592 | 0.7875 | 0.845 | 0.037-19.473 | 0.9160 | 1 | 0.00 | 346 | 1.00 | NA | NA | NA |
| 4th quartile | 7 | 0.00 | 1646 | 1.00 | 3.995 | 0.819-19.487 | 0.0867 | 5585 | 0.874-35.692 | 0.0692 | 3 | 0.00 | 674 | 1.00 | NA | NA | NA |
| Medical aid | 3 | 0.00 | 778 | 1.00 | 0.612 | 0.052-7.229 | 0.6969 | NA | NA | NA | 1 | 0.00 | 401 | 1.00 | NA | NA | NA |
| Hospital type | Interaction: NA | | | | | | | | | | Interaction: NA | | | | | | |
| General hospital | 22 | 0.01 | 4014 | 0.99 | 1.568 | 0.637-3.859 | 0.3272 | 1.651 | 0.608-4.481 | 0.3250 | 10 | 0.01 | 1697 | 0.99 | 2.153 | 0.535-8.669 | 0.2804 |
| Hospital | 0 | 0.00 | 251 | 1.00 | NA | NA | NA | NA | NA | NA | 0 | 0.00 | 134 | 1.00 | NA | NA | NA |
| Clinic | 1 | 0.00 | 393 | 1.00 | NA | NA | NA | NA | NA | NA | 1 | 0.00 | 257 | 1.00 | NA | NA | NA |
| Others | 0 | 0.00 | 18 | 1.00 | NA | NA | NA | NA | NA | NA | 0 | 0.00 | 7 | 1.00 | NA | NA | NA |
| History of COPD exacerbation | Interaction: 0.916 | | | | | | | | | | Interaction: 0.882 | | | | | | |
| None | 16 | 0.00 | 3940 | 1.00 | 1.905 | 0.681-5.326 | 0.2191 | 2.071 | 0.666-6.444 | 0.2086 | 8 | 0.00 | 1643 | 1.00 | 3.475 | 0.650-18.567 | 0.1451 |
| 1 moderate | 5 | 0.02 | 315 | 0.98 | 1.346 | 0.137-13.276 | 0.7990 | 0.033 | 0.000-309.903 | 0.4641 | 1 | 0.01 | 144 | 0.99 | NA | NA | NA |
| ≥ 2 moderate OR ≥ 1 severe | 2 | 0.00 | 421 | 1.00 | 2.138 | 0.207-22.067 | 0.5234 | NA | NA | NA | 2 | 0.01 | 208 | 0.99 | 1.483 | 0.074-29.924 | 0.7970 |
| History of pneumonia | Interaction: 0.889 | | | | | | | | | | Interaction: 0.993 | | | | | | |
| No | 20 | 0.00 | 4218 | 1.00 | 1.740 | 0.686-4.412 | 0.2431 | 1.968 | 0.712-5.438 | 0.1916 | 10 | 0.01 | 1821 | 0.99 | 1.889 | 0.517-6.901 | 0.3358 |
| Yes | 3 | 0.01 | 458 | 0.99 | 1.365 | 0.231-8.057 | 0.7315 | NA | NA | NA | 1 | 0.01 | 174 | 0.99 | NA | NA | NA |
| mCCI | Interaction: 0.905 | | | | | | | | | | Interaction: 0.968 | | | | | | |
| 0,1 | 9 | 0.00 | 2685 | 1.00 | 1.484 | 0.372-5.928 | 0.5761 | 0.880 | 0.178-4.361 | 0.8757 | 4 | 0.00 | 1146 | 1.00 | 1.386 | 0.137-14.006 | 0.7818 |
| 2 | 5 | 0.01 | 562 | 0.99 | 0.888 | 0.101-7.836 | 0.9146 | 16.545 | 0.332-824.774 | 0.1594 | 2 | 0.01 | 234 | 0.99 | 0.914 | 0.057-14.622 | 0.9494 |
| 3 | 4 | 0.01 | 776 | 0.99 | 4.629 | 0.617-34.696 | 0.1360 | NA | NA | NA | 3 | 0.01 | 309 | 0.99 | 3.382 | 0.362-31.570 | 0.2850 |
| 4+ | 5 | 0.01 | 653 | 0.99 | 2.013 | 0.343-11.818 | 0.4386 | 1.217 | 0.041-35.906 | 0.9094 | 2 | 0.01 | 306 | 0.99 | NA | NA | NA |
| mCCI categoty |  | | | | | | | | | |  | | | | | | |
| Congestive heart failure | Interaction: 0.996 | | | | | | | | | | Interaction: NA | | | | | | |
| No | 19 | 0.00 | 4221 | 1.00 | 2.369 | 0.939-5.977 | 0.0678 | 2.202 | 0.808-6.000 | 0.1228 | 11 | 0.01 | 1788 | 0.99 | 2.481 | 0.651-9.452 | 0.1829 |
| Yes | 4 | 0.01 | 455 | 0.99 | NA | NA | NA | NA | NA | NA | 0 | 0.00 | 207 | 1.00 | NA | NA | NA |
| Dementia | Interaction: 0.997 | | | | | | | | | | Interaction: NA | | | | | | |
| No | 22 | 0.00 | 4512 | 1.00 | 1.878 | 0.779-4.526 | 0.1601 | 2.391 | 0.911-6.276 | 0.0766 | 11 | 0.01 | 1925 | 0.99 | 2.446 | 0.646-9.258 | 0.1878 |
| Yes | 1 | 0.01 | 164 | 0.99 | NA | NA | NA | NA | NA | NA | 0 | 0.00 | 70 | 1.00 | NA | NA | NA |
| Chronic pulmonary disease | Interaction 0.924 | | | | | | | | | | Interaction: 0.406 | | | | | | |
| No | 10 | 0.00 | 2076 | 1.00 | 1.705 | 0.472-6.156 | 0.4156 | 2.376 | 0.501-11.263 | 0.2757 | 5 | 0.01 | 884 | 0.99 | 4.373 | 0.464-41.257 | 0.1975 |
| Yes | 13 | 0.00 | 2600 | 1.00 | 1.954 | 0.554-6.890 | 0.2975 | 1.408 | 0.368-5.391 | 0.6177 | 6 | 0.01 | 1111 | 0.99 | 2.095 | 0.258-17.000 | 0.4886 |
| Rheumatologic disease | Interaction: 0.998 | | | | | | | | | | Interaction: NA | | | | | | |
| No | 22 | 0.00 | 4506 | 1.00 | 1.903 | 0.790-4.579 | 0.1512 | 2.391 | 0.911-6.276 | 0.0766 | 11 | 0.01 | 1892 | 0.99 | 2.432 | 0.643-9.194 | 0.1903 |
| Yes | 1 | 0.01 | 170 | 0.99 | NA | NA | NA | NA | NA | NA | 0 | 0.00 | 103 | 1.00 | NA | NA | NA |
| Mild liver disease | Interaction: 0.844 | | | | | | | | | | Interaction: 0.873 | | | | | | |
| No | 18 | 0.00 | 3702 | 1.00 | 1.582 | 0.586-4.272 | 0.3653 | 2.348 | 0.761-7.247 | 0.1378 | 8 | 0.01 | 1576 | 0.99 | 2.259 | 0.442-11.542 | 0.3273 |
| Yes | 5 | 0.01 | 974 | 0.99 | 2.629 | 0.454-15.216 | 0.2806 | NA | NA | NA | 3 | 0.01 | 419 | 0.99 | 3.266 | 0.523-20.396 | 0.2054 |
| Diabetes with chronic complications | Interaction: 0.994 | | | | | | | | | | Interaction: NA | | | | | | |
| No | 20 | 0.00 | 4208 | 1.00 | 2.216 | 0.894-5.495 | 0.0858 | 3.078 | 1.140-8.309 | 0.0265 | 11 | 0.01 | 1792 | 0.99 | 2.536 | 0.665-9.674 | 0.1732 |
| Yes | 3 | 0.01 | 468 | 0.99 | NA | NA | NA | NA | NA | NA | 0 | 0.00 | 203 | 1.00 | NA | NA | NA |
| Hemiplegia or paraplegia | Interaction: 1.000 | | | | | | | | | | Interaction: NA | | | | | | |
| No | 23 | 0.00 | 4624 | 1.00 | 1.745 | 0.731-4.165 | 0.2095 | 1.902 | 0.736-4.912 | 0.1843 | 11 | 0.01 | 1975 | 0.99 | 2.436 | 0.644-9.205 | 0.1894 |
| Yes | 0 | 0.00 | 52 | 1.00 | NA | NA | NA | NA | NA | NA | 0 | 0.00 | 20 | 1.00 | NA | NA | NA |
| Renal disease | Interaction: 0.699 | | | | | | | | | | Interaction: 0.993 | | | | | | |
| No | 21 | 0.00 | 4548 | 1.00 | 1.647 | 0.660-4.109 | 0.2849 | 1.897 | 0.700-5.141 | 0.2082 | 10 | 0.01 | 1920 | 0.99 | 2.075 | 0.520-8.278 | 0.3013 |
| Yes | 2 | 0.02 | 128 | 0.98 | 2.446 | 0.154-38.748 | 0.5257 | NA | NA | NA | 1 | 0.01 | 75 | 0.99 | NA | NA | NA |
| Any malignancy, including lymphoma and leukemia | Interaction: 0.071 | | | | | | | | | | Interaction: 0.355 | | | | | | |
| No | 16 | 0.00 | 4053 | 1.00 | 1.102 | 0.353-3.436 | 0.8672 | 0.969 | 0.253-3.714 | 0.9632 | 6 | 0.00 | 1733 | 1.00 | 1.500 | 0.255-8.819 | 0.6539 |
| Yes | 7 | 0.01 | 623 | 0.99 | 4.524 | 1.013-20.205 | 0.0481 | 16.560 | 0.335-819.513 | 0.1585 | 5 | 0.02 | 262 | 0.98 | 4.297 | 0.481-38.413 | 0.1921 |
| Moderate or severe liver disease | Interaction: 0.999 | | | | | | | | | | Interaction: NA | | | | | | |
| No | 22 | 0.00 | 4636 | 1.00 | 1.865 | 0.771-4.508 | 0.1665 | 2.036 | 0.777-5.335 | 0.1478 | 11 | 0.01 | 1972 | 0.99 | 2.426 | 0.642-9.169 | 0.1913 |
| Yes | 1 | 0.02 | 40 | 0.98 | NA | NA | NA | NA | NA | NA | 0 | 0.00 | 23 | 1.00 | NA | NA | NA |
| Metastatic solid tumor | Interaction: 0.995 | | | | | | | | | | Interaction: 0.993 | | | | | | |
| No | 22 | 0.00 | 4617 | 1.00 | 1.544 | 0.619-3.851 | 0.3518 | 1.690 | 0.631-4.527 | 0.2964 | 10 | 0.01 | 1964 | 0.99 | 2.122 | 0.529-8.508 | 0.2881 |
| Yes | 1 | 0.02 | 59 | 0.98 | NA | NA | NA | NA | NA | NA | 1 | 0.03 | 31 | 0.97 | NA | NA | NA |
| HIV | Interaction: NA | | | | | | | | | | Interaction: NA | | | | | | |
| No | 23 | 0.00 | 4675 | 1.00 | 1.753 | 0.734-4.185 | 0.2060 | 1.902 | 0.736-4.912 | 0.1843 | 11 | 0.01 | 1995 | 0.99 | 2.432 | 0.643-9.197 | 0.1902 |
| Yes | 0 | 0.00 | 1 | 1.00 | NA | NA | NA | NA | NA | NA | NA | NA | NA | NA | NA | NA | NA |
| Index year | Interaction: NA | | | | | | | | | | Interaction: NA | | | | | | |
| 2005 | 2 | 0.01 | 360 | 0.99 | NA | NA | NA | NA | NA | NA | 1 | 0.01 | 160 | 0.99 | NA | NA | NA |
| 2006 | 3 | 0.01 | 303 | 0.99 | 7.646 | 0.801-72.945 | 0.0771 | NA | NA | NA | 2 | 0.01 | 228 | 0.99 | NA | NA | NA |
| 2007 | 2 | 0.01 | 361 | 0.99 | NA | NA | NA | NA | NA | NA | 1 | 0.00 | 211 | 1.00 | NA | NA | NA |
| 2008 | 4 | 0.01 | 449 | 0.99 | 1.741 | 0.176-17.169 | 0.6351 | 1.218 | 0.045-32.735 | 0.9066 | 3 | 0.02 | 155 | 0.98 | 0.555 | 0.042-7.251 | 0.6533 |
| 2009 | 0 | 0.00 | 490 | 1.00 | NA | NA | NA | NA | NA | NA | 0 | 0.00 | 220 | 1.00 | NA | NA | NA |
| 2010 | 3 | 0.01 | 582 | 0.99 | NA | NA | NA | NA | NA | NA | 1 | 0.00 | 201 | 1.00 | NA | NA | NA |
| 2011 | 1 | 0.00 | 565 | 1.00 | NA | NA | NA | NA | NA | NA | 0 | 0.00 | 214 | 1.00 | NA | NA | NA |
| 2012 | 2 | 0.00 | 543 | 1.00 | NA | NA | NA | NA | NA | NA | 0 | 0.00 | 208 | 1.00 | NA | NA | NA |
| 2013 | 0 | 0.00 | 447 | 1.00 | NA | NA | NA | NA | NA | NA | 0 | 0.00 | 159 | 1.00 | NA | NA | NA |
| 2014 | 5 | 0.01 | 460 | 0.99 | 5.818 | 0.973-34.795 | 0.0536 | NA | NA | NA | 3 | 0.02 | 188 | 0.98 | NA | NA | NA |
| 2015 | 1 | 0.01 | 116 | 0.99 | NA | NA | NA | NA | NA | NA | 0 | 0.00 | 51 | 1.00 | NA | NA | NA |

Abbreviations: HR, hazard ratio; CI, chronic obstructive pulmonary disease; COPD, modified Charlson comorbidity index; mCCI, human immunodeficiency virus; HIV, not applicable; NA.

**Table S13**. Pneumonia-associated risk factors

|  | Unmatched population | | | | | | Propensity score-matched population | | | | | |
| --- | --- | --- | --- | --- | --- | --- | --- | --- | --- | --- | --- | --- |
|  | Crude HR | | | Adjusted HR | | | Crude HR | | | Adjusted HR | | |
|  | HR | 95% CI | p-value | HR | 95% CI | p-value | HR | 95% CI | p-value | HR | 95% CI | p-value |
| Index med |  |  |  |  |  |  |  |  |  |  |  |  |
| LAMA | ref | - | - | Ref | - | - | ref | - | - | ref | - | - |
| ICS/LABA with fluticasone | 1.622 | 1.384-1.901 | <.0001 | 1.635 | 1.388-1.926 | <.0001 | 1.318 | 0.999-1.739 | 0.0507 | 1.496 | 1.204-1.859 | 0.0003 |
| ICS/LABA without fluticasone | 1.277 | 0.942-1.732 | 0.1150 | 1.232 | 0.906-1.675 | 0.1836 | 1.120 | 0.653-1.921 | 0.6804 | 1.002 | 0.682-1.473 | 0.9914 |
| ICS/LABA | 1.550 | 1.336-1.798 | <.0001 | 1.551 | 1.331-1.809 | <.0001 | 1.374 | 1.116-1.692 | 0.0028 | 1.389 | 1.127-1.713 | 0.0021 |
| Age |  |  |  |  |  |  |  |  |  |  |  |  |
| 55 to < 75 | ref | - | - | ref | - | - | ref | - | - |  |  |  |
| 75+ | 1.495 | 1.294-1.727 | <.0001 | 1.431 | 1.235-1.657 | <.0001 | 0.914 | 0.630-1.326 | 0.6354 |  |  |  |
| Sex |  |  |  |  |  |  |  |  |  |  |  |  |
| Male | 0.912 | 0.763-1.090 | 0.3095 |  |  |  | 0.727 | 0.430-1.230 | 0.2352 |  |  |  |
| Female | ref | - | - |  |  |  | ref | - | - |  |  |  |
| Income level |  |  |  |  |  |  |  |  |  |  |  |  |
| 1st quartile | ref | - | - | ref | - | - | ref | - | - |  |  |  |
| 2nd quartile | 1.168 | 0.893-1.528 | 0.2564 | 1.175 | 0.898-1.537 | 0.2402 | 1.279 | 0.623-2.626 | 0.5030 |  |  |  |
| 3rd quartile | 1.118 | 0.869-1.439 | 0.3850 | 1.140 | 0.886-1.468 | 0.3086 | 1.048 | 0.532-2.062 | 0.8928 |  |  |  |
| 4th quartile | 1.185 | 0.944-1.488 | 0.1435 | 1.150 | 0.914-1.446 | 0.2336 | 0.950 | 0.508-1.776 | 0.8725 |  |  |  |
| Medical aid | 1.575 | 1.235-2.010 | 0.0003 | 1.464 | 1.143-1.875 | 0.0025 | 1.005 | 0.511-1.977 | 0.9882 |  |  |  |
| Hospital type |  |  |  |  |  |  |  |  |  |  |  |  |
| General hospital | 1.400 | 1.073-1.828 | 0.0132 | 1.337 | 1.022-1.751 | 0.0344 | 1.844 | 0.902-3.767 | 0.0934 | 1.331 | 0.885-2.003 | 0.1698 |
| Hospital | 1.284 | 0.864-1.907 | 0.2165 | 1.117 | 0.751-1.661 | 0.5859 | 0.821 | 0.291-2.315 | 0.7085 | 0.823 | 0.449-1.511 | 0.5305 |
| Clinic | ref | - | - | ref | - | - | ref | - | - | ref | - | - |
| Others | 0.908 | 0.222-3.713 | 0.8934 | 0.640 | 0.156-2.632 | 0.5363 | 1.230 | 0.065-23.221 | 0.8902 | 1.182 | 0.18-9.867 | 0.8695 |
| History of pneumonia |  |  |  |  |  |  |  |  |  |  |  |  |
| No | ref | - | - | ref | - | - | ref | - | - | ref | - | - |
| Yes | 2.479 | 2.074-2.963 | <.0001 | 2.400 | 1.978-2.911 | <.0001 | 2.333 | 1.274-4.272 | 0.0060 | 2.123 | 1.580-2.852 | <.0001 |
| History of COPD exacerbation |  |  |  |  |  |  |  |  |  |  |  |  |
| None | ref | - | - | ref | - | - | ref | - | - |  |  |  |
| 1 moderate | 1.429 | 1.111-1.839 | 0.0055 | 1.240 | 0.950-1.619 | 0.1137 | 1.302 | 0.888-1.909 | 0.1766 |  |  |  |
| ≥ 2 moderate OR ≥ 1 severe | 1.574 | 1.279-1.936 | <.0001 | 1.083 | 0.860-1.364 | 0.4955 | 1.446 | 1.079-1.939 | 0.0137 |  |  |  |
| mCCI |  |  |  |  |  |  |  |  |  |  |  |  |
| 0, 1 | ref | - | - | ref | - | - | ref | - | - |  |  |  |
| 2 | 0.793 | 0.623-1.009 | 0.0594 | 0.815 | 0.639-1.040 | 0.0996 | 1.051 | 0.578-1.913 | 0.8696 |  |  |  |
| 3 | 1.064 | 0.881-1.283 | 0.5208 | 0.964 | 0.792-1.174 | 0.7169 | 0.972 | 0.552-1.710 | 0.9204 |  |  |  |
| 4+ | 1.230 | 1.012-1.496 | 0.0375 | 1.007 | 0.818-1.240 | 0.9498 | 0.871 | 0.533-1.421 | 0.5800 |  |  |  |
| mCCI category |  |  |  |  |  |  |  |  |  |  |  |  |
| Congestive heart failure |  |  |  |  |  |  |  |  |  |  |  |  |
| No | ref | - | - |  |  |  | ref | - | - |  |  |  |
| Yes | 1.053 | 0.839-1.322 | 0.6548 |  |  |  | 0.885 | 0.505-1.551 | 0.6688 |  |  |  |
| Dementia |  |  |  |  |  |  |  |  |  |  |  |  |
| No | ref | - | - |  |  |  | ref | - | - |  |  |  |
| Yes | 1.160 | 0.805-1.671 | 0.4251 |  |  |  | 1.000 | 0.375-2.664 | 1.0000 |  |  |  |
| Chronic pulmonary disease |  |  |  |  |  |  |  |  |  |  |  |  |
| No | ref | - | - | ref | - | - | ref | - | - |  |  |  |
| Yes | 1.223 | 1.063-1.407 | 0.0049 | 1.106 | 0.939-1.302 | 0.2273 | 1.160 | 0.795-1.693 | 0.4418 |  |  |  |
| Rheumatologic disease |  |  |  |  |  |  |  |  |  |  |  |  |
| No | ref | - | - |  |  |  | ref | - | - |  |  |  |
| Yes | 1.232 | 0.878-1.730 | 0.2282 |  |  |  | 0.917 | 0.405-2.078 | 0.8350 |  |  |  |
| Mild liver disease |  |  |  |  |  |  |  |  |  |  |  |  |
| No | ref | - | - |  |  |  | ref | - | - |  |  |  |
| Yes | 1.058 | 0.895-1.250 | 0.5101 |  |  |  | 0.814 | 0.521-1.272 | 0.3659 |  |  |  |
| Diabetes with chronic complications |  |  |  |  |  |  |  |  |  |  |  |  |
| No | ref | - | - |  |  |  | ref | - | - |  |  |  |
| Yes | 1.037 | 0.825-1.303 | 0.5101 |  |  |  | 0.724 | 0.413-1.270 | 0.2600 |  |  |  |
| Hemiplegia or paraplegia |  |  |  |  |  |  |  |  |  |  |  |  |
| No | ref | - | - |  |  |  | ref | - | - |  |  |  |
| Yes | 1.112 | 0.576-2.145 | 0.7521 |  |  |  | 1.500 | 0.251-8.977 | 0.6569 |  |  |  |
| Renal disease |  |  |  |  |  |  |  |  |  |  |  |  |
| No | ref | - | - |  |  |  | ref | - | - |  |  |  |
| Yes | 1.021 | 0.655-1.592 | 0.9257 |  |  |  | 1.125 | 0.434-2.915 | 0.8087 |  |  |  |
| Any malignancy, including lymphoma and leukemia |  |  |  |  |  |  |  |  |  |  |  |  |
| No | ref | - | - |  |  |  | ref | - | - |  |  |  |
| Yes | 1.032 | 0.843-1.264 | 0.7589 |  |  |  | 1.226 | 0.763-1.970 | 0.4002 |  |  |  |
| Moderate or severe liver disease |  |  |  |  |  |  |  |  |  |  |  |  |
| No | ref | - | - | ref | - | - | ref | - | - |  |  |  |
| Yes | 1.985 | 1.095-3.601 | 0.0240 | 1.916 | 1.030-3.567 | 0.0401 | 1.667 | 0.398-6.974 | 0.4843 |  |  |  |
| Metastatic solid tumor |  |  |  |  |  |  |  |  |  |  |  |  |
| No | ref | - | - |  |  |  | ref | - | - |  |  |  |
| Yes | 1.055 | 0.582-1.912 | 0.8604 |  |  |  | 1.834 | 0.254-2.732 | 0.7640 |  |  |  |
| HIV |  |  |  |  |  |  |  |  |  |  |  |  |
| No | ref | - | - |  |  |  | NA | NA | NA |  |  |  |
| Yes | NA | NA | NA |  |  |  | NA | NA | NA |  |  |  |

Abbreviations: HR, hazard ratio; CI, chronic obstructive pulmonary disease; COPD, modified Charlson comorbidity index; mCCI, human immunodeficiency virus; HIV, not applicable; NA.
